# Cytotoxic CD4 T Cells—Friend or Foe during Viral Infection?

**DOI:** 10.3389/fimmu.2017.00019

**Published:** 2017-01-23

**Authors:** Jennifer A. Juno, David van Bockel, Stephen J. Kent, Anthony D. Kelleher, John J. Zaunders, C. Mee Ling Munier

**Affiliations:** ^1^Department of Microbiology and Immunology, Peter Doherty Institute, University of Melbourne, Melbourne, VIC, Australia; ^2^Immunovirology and Pathogenesis Program, The Kirby Institute for Infection and Immunity in Society, University of New South Wales Australia, Sydney, NSW, Australia; ^3^Melbourne Sexual Health Centre, Department of Infectious Diseases, Alfred Health, Central Clinical School, Monash University, Melbourne, VIC, Australia; ^4^ARC Centre of Excellence in Convergent Bio-Nano Science and Technology, University of Melbourne, Parkville, VIC, Australia; ^5^St Vincent’s Hospital, Sydney, NSW, Australia

**Keywords:** CD4, cytotoxic, perforin, granzyme, HIV, CMV, EBV, influenza

## Abstract

CD4 T cells with cytotoxic function were once thought to be an artifact due to long-term *in vitro* cultures but have in more recent years become accepted and reported in the literature in response to a number of viral infections. In this review, we focus on cytotoxic CD4 T cells in the context of human viral infections and in some infections that affect mice and non-human primates. We examine the effector mechanisms used by cytotoxic CD4 cells, the phenotypes that describe this population, and the transcription factors and pathways that lead to their induction following infection. We further consider the cells that are the predominant targets of this effector subset and describe the viral infections in which CD4 cytotoxic T lymphocytes have been shown to play a protective or pathologic role. Cytotoxic CD4 T cells are detected in the circulation at much higher levels than previously realized and are now recognized to have an important role in the immune response to viral infections.

## Introduction

It is well established that CD4 T cells play a significant, often central, role in the immune response to viral infections. CD4 T cells provide both “help” to enable B cells to generate effective neutralizing antibodies through affinity maturation and antibody class switching, as well as promoting the development and maintenance of cytotoxic virus-specific CD8 T cell responses. CD4 T cells have an ability to be functional memory cells and can themselves have a direct antiviral effect, although this is a lesser known role. Between 1977 and 2001, CD4 T cells with cytotoxic characteristics were described sporadically in the literature.

Initial reports of CD4 T cells with cytotoxic characteristics from cultured CD4 T cell lines and clones (1–5) were met with doubt as to whether these CD4 T cells were in fact true cytotoxic T lymphocytes (CTL), or an anomaly owing to long-term *in vitro* cultures (6). Only one report described human Leu3a+ CTL in PBMC, prior to the introduction of the CD4 nomenclature (7). The Leu3a antibody is CD4 specific, so that this report described Cytomegalovirus (CMV)-specific “helper” cells with an *ex vivo* CTL function, prior to the introduction of the CD nomenclature. From 2001, the ability of human CD4 to function as CTL *ex vivo* began to be more widely reported (8–13). Further, there is increasing evidence that cytolytic CD4 T cells (CD4 CTL) are detected following vaccinations, including against smallpox (14, 15), poliovirus (16), and in response to the vaccines (ALVAC/AIDSVAX) given in the RV144 HIV vaccine study (17).

Herein, we review the characteristics of CD4 CTL across a range of human viral infections including human immunodeficiency virus type 1 (HIV-1) (9–11, 18–20), CMV (8, 10, 12, 21, 22), Epstein–Barr virus (EBV) (23–25), influenza (26, 27), viral hepatitis (28), hantavirus (29), dengue (30–33), and parvovirus B19 (34). CD4 CTL may also be involved more broadly in the regulation of immune responses, through regulatory T cell (Treg) function (to be discussed later) and may also be involved in other non-viral infections and anti-tumor responses. Clearly, these cells represent an additional mechanism by which CD4 T cells contribute generally to human immunity, and below we concentrate on antiviral immunity.

## Cytotoxic Effector Mechanisms

### CD4 Cytotoxicity *via* Fas Ligand

CD4 CTL utilize two fundamental cytotoxic effector mechanisms used also by CD8 CTL and natural killer (NK) cells. The first is the Fas/Fas ligand-mediated pathway, which involves binding of the cell surface Fas ligand (FasL; CD95L; CD178) expressed on the effector cells binding to its cognate receptor Fas (CD95) expressed on the target cells. Trimerization of Fas on the target cell leads to recruitment of the intracellular FADD/caspase 8/c-FLIP death-inducing signaling complex, and finally to caspase 3-mediated apoptotic cell death (35–37).

### CD4 Cytotoxicity *via* Perforin and Granzymes

The second major mechanism of cytotoxicity is the directed exocytosis of specialized granules into target cells to induce apoptosis [reviewed in Ref. (38)]. Cytotoxic granules were originally characterized in CD8 CTL and NK cells as large vesicles, which in turn contain numerous smaller internal vesicles and an electron dense core (39). Cytotoxic granules undergo exocytosis after specific T cell receptor (TCR) signaling; a key regulator of this process is Rab27a. Genetic defects in Rab27a result in Griscelli syndrome type 2 [reviewed in Ref. (40)] an autosomal recessive disorder of pigmentation and severe immune deficiency (41). The pore-forming protein perforin is the best-described cytotoxic molecule in these granules (42, 43); it enables direct transfer of cytotoxic molecules such as granzymes and granulysin into target cells. There are five known granzymes or serine proteases in humans (A, B, H, K, and M) with various substrate specificities [reviewed in Ref. (44–47)]. Granzyme (Gzm) A and GzmB are the most extensively studied and are the most abundant in cytotoxic granules (48, 49), while the other granzymes H, K, and M are less well understood. GzmA and GzmK genes are located on chromosome 5 in humans and on chromosome 13 in mice [reviewed in Ref. (50)], and while both have tryptase-like activity, there is only partial overlap of substrates *in vivo* (51). In contrast, GzmB and GzmH (GzmC in mice) are chymases, with genes located on chromosome 14 in humans and mice [reviewed in Ref. (44)].

Given the well-defined nature of CD8 CTL in comparison to CD4 CTL, comparisons of the cytolytic machinery of both T cell subsets can further our understanding of the relative impact of CD4 cytolytic activity in infection. In a recent murine study of lymphocytic choriomeningitis virus, Hildemann et al. used an *in vivo* CTL assay to demonstrate that CD4 CTL were readily generated and had comparable CTL activity to CD8 CTL when factors such as effector to target ratios were adjusted (52). A difference they noted between CD4 and CD8 CTL killing was the slightly delayed killing kinetics of the CD4 CTL (52). Another comparison showed that CD8 CTL stored more intracellular GzmB than CD4 T cells; however, secretion of GzmB was equivalent between both T cell subsets (although CD8 T cells secreted more perforin than CD4) (53). In our recent study of activated CD4 and CD8 T cells responding to primary HIV-1 infection and vaccination with vaccinia virus, we confirmed that CD8 T cells express significantly higher levels of perforin and T cell intracellular antigen, TIA-1 [gene name *nkg7*, also known as granule membrane protein, GMP-17 (54)] compared to CD4 T cells (15). Despite these apparent differences in the carriage of cytolytic machinery, it would appear from the above studies that the antiviral activity of CD4 and CD8 CTL is similar.

## Phenotype of CD4 CTL in Healthy Adults

Perforin-expressing CD4 T cells identified *ex vivo* in peripheral blood from healthy humans have a distinct cell surface phenotype. These cells in healthy adults are typically CD45RO+, highly express the integrins CD11a and CD11b, and do not express the costimulatory receptors CD27 or CD28, or the chemokine receptor CCR7 (9). CD4 CTL detected in healthy human blood are not activated and are not undergoing proliferation, as they are CD38low, CD69neg, Ki67neg, and Bcl-2high (9). Also, CD4 CTL are distinct from NK T cells as they do not express CD16, CD56, or CD161 (9). CD57 expression, a marker of terminal differentiation (55), appears to be upregulated on CD4 CTL (56).

Another marker associated with CD4 CTL is Fc receptor-like 6 (FCRL6); Schreeder et al. found that FCRL6+ CD4 T cells also expressed perforin, CD57, and NKG2D (57). NKG2D, originally identified on NK cells, is a killer lectin-like receptor. Following ligation, NKG2D initiates an intracellular cascade leading to perforin exocytosis and consequently cytotoxicity. Expression of NKG2D on CD4 T cells has been suggested to occur following repeated exposures to antigen and is significantly increased in elderly (mean age: 84.3 years) compared to young (mean age: 39 years) adults (58). In contrast to CD28+ NKG2D+ CD4 T cells, their CD28null counterparts express perforin and GzmB and have a more differentiated phenotype (58). Hence, the overall phenotype of CD4 CTL in healthy adults indicate that these cells are at an advanced stage of cellular differentiation, consistent with the suggestion that these cells are generated in the presence of chronic antigen exposure (56, 59, 60). However, we and others have shown that CD4 CTL are also detected in the primary response to acute viral infections, which will be discussed later in this review. Table 1 provides a summary of the CD4 CTL phenotypes described in healthy adults, non-human primates, and mice in response to various viral infections.

**Table 1:** **Summary of known CD4 cytotoxic T lymphocyte (CTL) phenotype and mechanism of cytolysis in human, non-human primate (NHP), and murine models**.

	Model	Phenotype	Conditions	Reference
Healthy adults	Cell surface	CD11a/b+, CD27−, CD28− CD45RO+, CCR7−		Appay et al. (9)
	Activation	CD38lo, CD69−, Bcl2++, Ki67−		Appay (56)
	Differentiation	CD57+, perforin, GzmB		Appay et al. (9) and Appay (56)
	Effector	CD57+, FRCL6+, NKG2D+, perforin+		Schreeder et al. (57)
	Senescence	NKG2D+		Alonso-Arias et al. (58)

Regulatory T cells (Tregs)	Human	GzmB+	*In vitro* (αCD3, αCD28, IL-2)	Efimova and Kelley (106)

Tregs	Murine	GzmA+, GzmB+, perforin+	*In vitro* phenotype (effector CD4 T-cell+ IL-2++)	Czystowska et al. (111)

Infection/pathogen				

Human immunodeficiency virus type 1 (HIV-1)	Human	GzmA+, perforin+, TIA-1/GMP-17+		Appay et al. (9)

Acute HIV-1	Human	CD38+++, CD57−, Bcl2lo, IFN-γ, Ki67+, TIA-1+		Zaunders et al. (117)

Simian immunodeficiency virus	NHP	CD28+, CD45RA−, CD95+, CCR7−, GrzmB+		von Gegerfelt et al. (113)

Cytomegalovirus (CMV)	Human	CD28−, CD27−, GzmB+, perforin+	Therapy cessation	van Leeuwen et al. (12)
		CD27−, CD28−, perforin+	Latent CMV	Appay et al. (9)
		CD244+, CCR5+, GzmA+ IFN-γ+, TIA-1+	Latent CMV	Zaunders et al. (10) and Zaunders et al. (117)
		CD107a, GzmA+ GzmB+, IFN-γ+, MIP-1β+, perforin+, TNF+	Latent CMV	Casazza et al. (21)
		CX3CR1+, Gransulysin+, GzmA/B/H+, IFN-γ+, perforin+, TNF+	*In vitro* phenotype	Pachnio et al. (130)
		CD28−, CX3CR1+, NKG2D+, perforin+	Posttransplant	Shabir et al. (132)

Acute CMV	Human	CD27−, CD28+, IFN-γ+, GrzmB+, TNF+ (acute)		Gamadia et al. (128)

Epstein–Barr virus	Human	Eomes+Tbet+	*In vitro* phenotype (CD137)	Akhmetzyanova et al. (151)

Influenza	Human	Perforin+, GzmB+	Vaccine phenotype (BMDC, αCD3, IFN-γ, IL-2), *in vitro* phenotype	Zhou and McElhaney (126)

Influenza	Mouse	GzmB+	(CpG stimulus)	Vogel and Brown (164)
		GzmB+	(PR8)	Brown et al. (72)
		Perforin+, GzmB+	*In vitro* phenotype and tissue resident	Hua et al. (64)

Vaccinia	Human	CD4+ CD8− Leu11−		Littaua et al. (159)
				Demkowicz et al. (160)
		IFN-γ+ TIA-1+ CD57−	Vaccine phenotype	Zaunders et al. (14)
		GrzmA, GrzmK, KLRB1/CD161, Rab27a, granulysin, TIA-1, perforin	Microarray analysis	Munier et al. (15)

Ectromelia	Mouse	GzmB+		Fang et al. (157)

Hepatitis	Human	Perforin+	Hepatitis B virus	Aslan et al. (28)
		Perforin+	Hepatitis C virus	Aslan et al. (28)
		Perforin++	Hepatitis D virus	Aslan et al. (28)

Dengue	Human	Perforin+	Antigen-presenting cell targets	Gagnon et al. (77)
		Fas/FasL	HepG2 cells	Weiskopf et al. (155)
		CD45RA+ CCR6−, CCR7−, CCR4−, CXCR3−, CD8a+, CD107a+, Gzm+, Eomes+, CX3CR1+		

Parvovirus	Human	CD57+, GzmB+, perforin+, IL-17+	*In vitro* phenotype	Kumar et al. (34)

Hantavirus	Human	GzmB+, perforin+, CD107a±		Ma et al. (29)

Human papillomavirus	Human	CD28− NKG2D+ (CD107a and CD161 negatively correlated with frequency)		Garcia-Chagollan et al. (102)

*Parentheses () indicate the conditions applied to each model, which has been linked to phenotype*.

## Mechanisms Regulating CD4 CTL Development

### Transcription Factors Conferring Cytotoxicity

It remains a matter of debate whether CD4 CTL represent a novel CD4 T cell lineage, or simply a subset of cells that have acquired cytotoxic function in addition to conventional T helper characteristics. Detailed studies have established the role of a series of transcription factors (TFs) in regulating T helper fate (61), leading to the question of whether specific TFs are also required for CD4 CTL development. The T-box family TFs T-bet and Eomesodermin (Eomes) have been known for some time as master regulators of cytotoxicity in CD8 CTL (62, 63); recently, studies have confirmed a role for both molecules in regulating CD4 T cell cytotoxicity as well. While T-bet expression is required for the induction of the CD4 Th1 lineage and IFN-γ production, it can also bind to the promoters of *GzmB* and *Prf1* in both CD4 and CD8 T cells (64, 65). During influenza infection, the binding of T-bet to cytotoxic gene promoters in CD4 T cells is regulated by Blimp-1 expression *via* a mechanism involving IL-2, type I interferons, and STAT2 signaling (64). T-bet- or Blimp-1-deficient T cells display impaired GzmB expression *in vivo*, confirming the role of these TFs in CD4 CTL activity. T-bet is not, however, the only TF involved in conferring cytotoxicity on CD4 T cells. Qui et al. were the first to demonstrate a requirement for Eomes in GzmB expression by CD4 T cells (66), although they did not assess its contribution to other mechanisms of CD4 CTL killing. To specifically dissect how expression of Eomes contributes to the generation of CD4 CTL, Eshima et al. transfected two murine CD4 T cell lines with a single copy of Eomes and assessed the expression of both perforin/granzyme and Fas/FasL cytotoxic pathways (67). Transfection of Eomes resulted in the acquisition of IFN-γ expression, upregulation of FasL upon antigenic stimulation, and expression of perforin/granzyme that lead to acquisition of cytotoxic activity. Interestingly, expression of Eomes induced cytotoxicity more efficiently than transfection with the perforin gene, suggesting that Eomes confers cytotoxicity on T cells through additional mechanisms beyond the induction of perforin expression.

**Figure 1:**
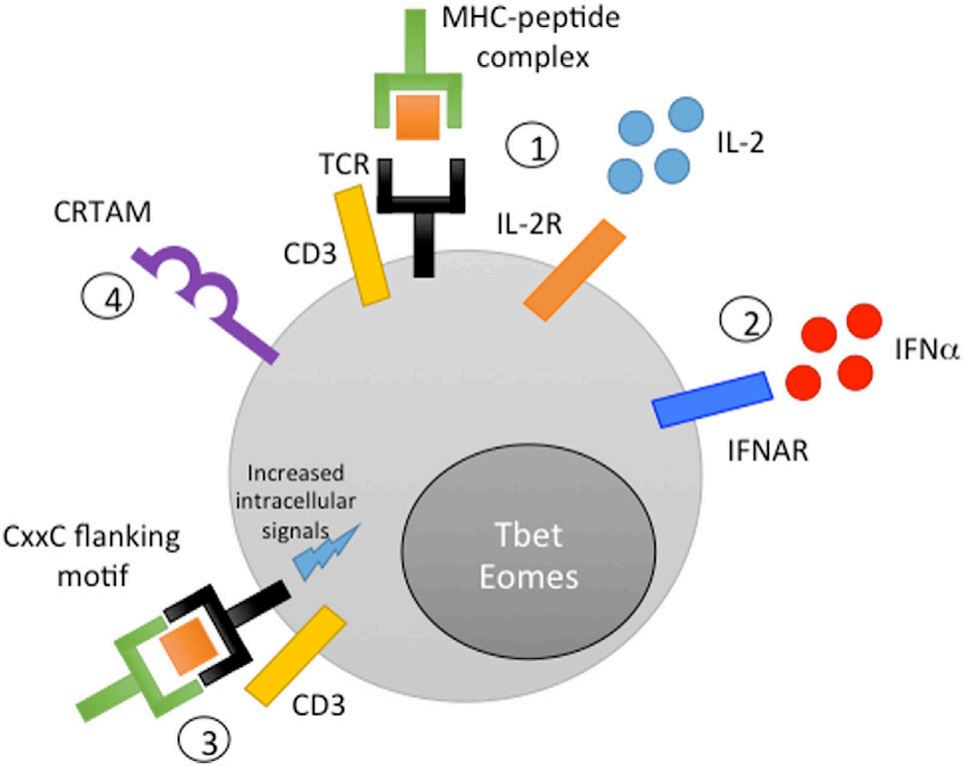
**Mechanisms of CD4 cytotoxic T lymphocyte (CTL) generation**. (1) Peptide stimulation in conjunction with IL-2 signaling induces CD4 CTL activity *in vitro*. (2) Interferon signaling or other inflammatory cytokine signals can synergize with or compensate for low IL-2 signaling to promote acquisition of cytolytic function. (3) The inclusion of a CxxC motif in flanking residues of the peptide antigen is thought to improve the strength of the immunological synapse and promote cytotoxicity. (4) Class I-restricted T cell-associated molecule-mediated intracellular signaling promotes Eomes expression and the acquisition of cytolytic activity.

A critical clue as to how Eomes is induced in CD4 CTL came from a new study by Takeuchi et al., who identified class I-restricted T cell-associated molecule (CRTAM) as a determinant of a putative CD4 CTL lineage in humans and mice (68). CRTAM, a surface membrane protein that binds the ligand cell adhesion molecule 1, is expressed on a portion of memory and naïve CD4 T cells following activation. CRTAM-expressing CD4 cells are capable of IFN-γ production under Th0 polarizing conditions, express elevated levels of Eomes and GzmB, and can differentiate into CD4 CTL *in vitro* (Figure 1). Intracellular signaling through the cytoplasmic domain of CRTAM is required for the expression of Eomes, IFN-γ, and GzmB/perforin in CRTAM+ cells, placing CRTAM upstream of Eomes in a signaling pathway that regulates CD4 CTL development. Interestingly, CRTAM+ CD4 cells are found preferentially in the lung and intestinal lamina propria, the same tissues in which CD4 CTL are efficiently generated during viral infections in murine models (discussed further below). The authors confirmed that viral infection increases the frequency of CRTAM+ cells in the lung and that CRTAM knock-out mice exhibit decreased CD4 CTL activity during infection. Overall, these results suggest that CRTAM+ CD4 T cells represent the precursor of CD4 CTL and identify CRTAM as both a useful marker for identifying potential CD4 CTL and a potential therapeutic target to modulate CD4 CTL activity *in vivo*.

While the majority of the studies described above focused on CD4 CTL isolated from peripheral blood or the lungs, Mucida et al. recently described the presence of CD4 CTL in the murine gut (69). In healthy mice, they identified a CD4 T cell population that did not express the master Th regulator ThPOK (or cKrox, encoded by *Zbtb7b*). ThPOK is responsible for inducing Th fate and repressing the expression of the CD8 CTL regulator Runx3 in thymocytes. As such, all CD4 T cells isolated from the spleen and lymph nodes of ThoPOK-reporter mice expressed ThPOK; in contrast, many CD4+ intraepithelial lymphocytes (IELs) were ThPOK− and CD8a+. These ThPOK− CD4 T cells phenotypically resembled CD8 CTL, including the expression of GzmB, CD107a, and LAMP-1, and were capable of killing *in vitro*. Interestingly, CRTAM expression was also detected on the ThPOK−CD8a+ CD4 CTL, providing additional evidence to support CRTAM as a marker of CD4 CTL in multiple tissues. The authors confirmed that the IEL CD4 CTL lost ThPOK expression post-thymically, differentiated into CD4 CTL in the context of chronic antigen exposure, and exhibited killing following antigen and IL-15 stimulation. Subsequent studies demonstrated that T-bet and Runx3 expression are responsible for driving the loss of ThPOK in IELs (70, 71). Overall, these data provide compelling evidence of CD4 T cell plasticity, and the ability of lineage-committed T helper cells to be re-directed toward cytotoxicity.

### Cytokine Microenvironment and Antigen Stimulation

The identification of CD4 CTL both in healthy humans and directly *ex vivo* following viral infection raises the question of how these cells are generated *in vivo*. Although this issue has been challenging to address, *in vitro* studies and insights from *in vivo* mouse experiments have provided clues to mechanisms that are likely also relevant in humans *in vivo* (Figure 1). Importantly, studies of influenza infection in mice have demonstrated that the generation of CD4 CTL does not necessarily require prolonged, chronic antigen stimulation, but can instead occur during acute infection (72, 73). Overall, it appears that the combination of a cytokine-mediated inflammatory signal and antigen presentation is required for CD4 T cells to acquire cytotoxicity (69, 74).

Although CD4 cytolytic activity is commonly observed in Th1 cells (75–77), CD4 CTL can be generated *in vitro* prior to full Th polarization and differentiation and do not require IFN-γ (78). IL-2, however, appears to be critical in the generation of CD4 CTL in both mice (64, 66, 79) and in *ex vivo* human peripheral blood samples (80, 81). In contrast, the addition of IL-4 to IL-2-stimulated cell culture inhibits the development of CD4 CTL in a dose-dependent manner. At sufficiently high antigen concentration, a low to intermediate level of cytotoxicity can be induced in the absence of IL-2, although this cytotoxic activity is primarily Fas/FasL mediated; exogenous IL-2 is required to induce perforin-mediated killing at all antigen concentrations (Figure 1). *In vivo*, it appears that the presence of inflammatory signals (such as pro-inflammatory cytokines or interferon signaling) during infection can compensate for, or synergize with, low IL-2 levels, leading to the generation of cytotoxic CD4 effectors. Type I interferons such as IFN-α signal through STAT2; STAT2-deficient mice exhibit significantly reduced GzmB expression in lung CD4 T cells following influenza infection, supporting the role of this pathway in generating CD4 CTL *in vivo*. Loss of interferon regulatory factor 3 also impairs GzmB expression and CD4 memory cells (82), providing further evidence for the involvement of interferon signaling in CTL induction. Workman et al. have also suggested that inflammatory cytokines such as IL-6 in the lung microenvironment may promote CD4 CTL activity, similar to the way in which CD8 CTL require signals from DCs and IL-15 during influenza infection (79). IL-15-responsiveness appears to be a trait shared by both CD4 and CD8 CTL; Mucida et al. showed that ThPOK− CD4 CTL in the murine gut remained quiescent when stimulated with only their cognate antigen, but exhibited cytotoxicity in the presence of antigen and IL-15 (69).

Modulation of antigen also impacts the functionality of CD4 CTL. Low levels of antigen generate the highest levels of cytotoxicity and produce CTL that retain the ability to express IL-5, IL-10, and IL-13; in contrast, CTL generated with higher levels of peptide produce almost exclusively IFN-γ and TNF (78). In addition to antigen concentration, characteristics of the cognate peptide can significantly impact the generation of CD4 CTL both *in vitro* and *in vivo* (83). Carlier et al. demonstrated, for several well-described epitopes in transgenic mice, that insertion of a CxxC motif into the flanking residues of a major histocompatibility complex (MHC) class II-restricted peptide results in acquisition of cytolytic activity by the antigen-specific CD4 T cell (Figure 1). This novel effector function is thought to occur due to an increased strength of the immunological synapse between the antigen-presenting cell (APC) and the antigen-specific CD4 T cell (84), leading to lck/ZAP70-mediated signaling, promoting T cell proliferation and expression of FasL and GzmB. Both naïve and polarized CD4 cells can acquire CTL activity through this mechanism, providing further evidence in agreement with Mucida et al. that T helper polarization is not a terminally differentiated state.

Given the particular requirements for inflammatory signaling and antigen presentation in CD4 CTL generation, it is perhaps unsurprising that some tissues appear to better promote cytolytic activity than others. During acute influenza infection, protective CD4 effectors appear to be specifically generated at the site of infection in the lung, where inflammatory signals and antigen are highly concentrated (74). Importantly, multiple studies have shown the presence of *in vivo*-generated GzmB+ CD4 CTL in the lung, but not the draining lymph nodes or spleen, at 7–8 days postinfection (64, 72). The microenvironment of the gut mucosa is similarly well suited to drive CD4 CTL differentiation, as the presence of local IFN-γ and IL-27 can promote T-bet expression (85). Unsurprisingly, delivery of antigen directly to mucosal tissues therefore appears to be an efficient method of eliciting CD4 CTL responses. Dutta et al. demonstrated that sterilizing immunity to homotypic influenza virus can be induced in mice through intranasal, but not intramuscular, inoculation of low-dose PR8 (86). In this model, intranasal infection was significantly more effective at inducing CD4 CTL activity in the lung than intramuscular infection, even at substantially lower infectious doses.

While these studies have provided substantial insight into the conditions under which CD4 CTL can be generated, the precise mechanisms associated with differentiation of CD4 CTL *in vivo* remain to be fully elucidated. New technology is now available to investigate single-cell transcriptomics, and future studies will likely identify transcription and repressor factors associated with typical CTL mRNAs such as for *nkg7* (TIA-1), perforin, and granzymes at the single-cell level.

## Antigen Recognition by CD4 CTL and Possible Targets

### MHC Class II-Expressing APCs

Targets of CD4 CTL must express the MHC class II molecules. Cells that constitutively express HLA class II include APCs such as dendritic cells, macrophages including tissue-resident macrophages such as Kupffer cells in the liver, alveolar macrophages in the lungs, and B cells. The question therefore arises as to whether CD4 CTL kill APCs during normal cognate antigen interaction. DC have been described as expressing SerpinB9, which is an inhibitor of GzmB (87), but whether this prevents cytotoxicity or is involved in cross-presentation to CD8 T cells is unknown (88). Nevertheless, CD4 CTL appear to be prominent in infections that typically target APC or B cells, particularly those mediated by CMV, EBV, and dengue virus (DENV), and also HIV-1 that targets HLA class II+ activated CD4 T cells themselves, as discussed below. There are well-characterized pathways for viral-derived peptides to be presented by HLA class I on the surface of infected cells to CD8 CTL, but presentation by HLA class II on infected cells is less well characterized. HLA class II presentation by professional APC typically involves specialized endocytosis and transfer of peptides to HLA class II molecules in loading vesicles. One possible pathway for loading of viral peptides on HLA class II in infected cells may involve autophagy, and this presentation may be enhanced by IFN-γ (89).

Regardless of whether APC killing is a common function of CD4 CTL during natural viral infection, manipulation of cytolytic CD4 responses may provide interesting new avenues for immunotherapy. Carlier et al. raised CD4 CTL *in vitro* against peptides modified to contain a CxxC motif and demonstrated that these CTL are capable of inducing apoptosis in APCs, even if the APCs are presenting the original, non-CxxC-containing peptide. This approach allows for the suppression of both DC- and B cell-mediated immune responses; crucially, these CD4 CTL are also capable of killing bystander CD4 T cells that have come into contact with and are activated by the same APC, allowing an antigen-specific CD4 CTL to suppress polyclonal T cells recognizing the same APC (84).

### MHC Class I-Expressing APCs

Surprisingly, cytolytic CD4 activity has been demonstrated to occur against myeloid APCs, in a MHC class I-dependent manner (90). This function is specifically carried out by type 1 regulatory (Tr1) cells, which are CD4+ Foxp3− regulatory cells that are induced in the context of chronic antigen stimulation and IL-10 production [recently reviewed in Ref. (91, 92)]. Recent identification of the Tr1 surface phentoype as CD4+ CD49b+ LAG-3+ CD226+ has greatly facilitated research into their functions (93). Tr1 cells generated *in vitro* express GzmB and perforin and are capable of killing target cells with substantially greater efficiency than other cytotoxic CD4 cells (94), possibly due to high levels of STAT3 phosphorylation (95). Magnani et al. demonstrated the perforin- and granzyme-mediated killing of myeloid APCs by Tr1 cells, which was dependent not only on HLA class I recognition but also CD2 expression and recognition of myeloid APC CD155/112 by Tr1-expressed CD226. As these cells are better defined, future studies will be able to provide a more comprehensive description of the role of cytotoxic Tr1 cells in suppressing and modulating the immune response during infection.

### Epithelial Cells and Other Non-APCs

Upregulation of MHC class II on murine lung epithelial cells, airway epithelial cells, or cell lines has been shown to occur following infection with Mycobacterium tuberculosis (96), influenza (72), and parainfluenza (97), respectively, as well as with IFN-γ treatment [reviewed in Ref. (13)], which is consistent with the ability of IFN-γ to upregulate MHC class II expression *via* JAK1/JAK2 activation (98). This upregulation occurs in both bone marrow-derived and non-bone marrow-derived cells (98). These observations highlight that infections with various pathogens lead to the upregulation of MHC class II expression on multiple cell types, allowing cells other than professional APCs to provide targets for CD4 CTL. During influenza infection, the trafficking and induction of CD4 CTL in the lung coincides with the expression of MHC class II molecules on lung epithelial cells at 5 days postinfection, which is likely critical for the contribution of CD4 CTL to protection from lethal infection (72). Similarly, hepatocytes are likely targets for CD4 CTL recognition during hepatitis infection, as these cells can express class II molecules and present antigen to virus-specific CD4 cells (99). A potential role for CD4 CTL activity in mediating immunopathology is supported by the observation that two patients who spontaneously cleared Hepatitis C virus (HCV) infection exhibited a significant decrease in perforin-expressing CD4 cells that coincided with viral RNA clearance.

### Virally Induced Tumors As Targets

Several viral infections are associated with the development of malignancies, including human papillomavirus (100) and EBV (discussed later in this review). Tumors expressing viral antigens can therefore provide important targets for CD4 CTL activity. Cervical intraepithelial neoplasia, a premalignant abnormal growth preceding cervical cancer, is negatively associated with the presence of circulating cytotoxic CD4 T cells (101). Garcia-Chagollan et al. identified a population of CD4+ CD28± NKG2D+ T-cells, which appear to be overrepresented in cervical cancer patients and which express the cytotoxic markers CD107a and CD161 (102). While the role of CD4 CTL in protection against tumor formation requires more directed study, the use of immunotherapy and vaccination to induce cytotoxic CD4 responses against tumor antigens is gaining interest, as discussed later in the review.

### Tregs and Their Targets

Regulatory T cells, similar to Tr1 cells, play important roles in the suppression and regulation of the immune response. Tregs are defined by the expression of Foxp3 and can exert regulatory function through both cytokine production (notably IL-10 and TGFβ) and contact-dependent mechanisms including CTLA-4 expression [reviewed by Arce-Sillas et al. (103)]. Nevertheless, the requirement for GzmB, but not perforin, in contact-mediated Treg suppression was clearly demonstrated in a mouse model (104) and further mouse studies demonstrated that GzmB expression in lung Tregs regulated cellular infiltration and inflammation during respiratory syncytial virus infection (105). The function and expression of granzyme and perforin in human Tregs, however, differs in some respects from the murine system. Efimova and Kelley reported that *ex vivo*, circulating human natural Tregs (nTregs) from healthy donors do not express GzmB and require both CD3/CD28 stimulation and IL-2 treatment to upregulate GzmB expression (106). GzmB induction was dependent on the mTOR and PI3K pathway. In direct contrast, however, Grossman et al. demonstrated that nTregs stimulated with anti-CD3/CD28 and IL-2 upregulated the expression of GzmA, but not GzmB, and killed target cells *via* a perforin-dependent pathway (107). Currently, the reasons for this discrepancy are unknown and unresolved; in mice, GzmA, but not GzmB, contributes to Treg function during graft-versus-host disease (108, 109), raising the possibility that human Tregs may selectively express GzmA and GzmB selectively under specific circumstances.

The induction of cytotoxicity by Tregs has several consequences. First, Tregs must avoid self-inflicted apoptosis, as the expression of GzmB by nTregs has the potential to induce Treg death and prevent the suppression of target cells. In mice, Tregs concurrently upregulate both GzmB and the endogenous inhibitor serine protease inhibitor 6, which hinders Treg apoptosis and promotes suppressive activity (110). Second, both Tregs and conventional CD4 CTL can exhibit cytotoxic responses against each other. When responder CD4 T cells and Treg are cocultured in the presence of IL-2, both cell populations can upregulate GzmA, GzmB, and perforin. At low concentrations of IL-2, Tregs became susceptible to responder cell cytotoxicity and were killed. At high concentrations of IL-2, however, the Tregs maintained their suppressive activity and induced apoptosis of the responder cells (111). A similar study, focused on an effector population of nTregs expressing HLA-DR, also demonstrated the capacity of responder CD4 cells to upregulate expression of GzmB after TCR stimulation and kill Tregs (112). Together, these studies demonstrate the myriad of pathways through which CD4 cytolytic activity can play either a suppressive function or promote escape from regulatory responses.

## Infections in Which CD4 CTL are Protective

### Simian Immunodeficiency Virus

Antiviral CD4 CTL have been found in the response to simian immunodeficiency virus (SIV) infection of rhesus macaques. SIV-infected macaques controlling viral replication were depleted of CD8 T cells, leading to increased viremia that was rapidly controlled despite the lack of SIV-specific CD8 T cells (113). Viral control was associated with antibody responses and expansion of circulating SIV-specific CD4 T cells that were CD45RA− CD28+ CD95+ CCR7− and expressed GzmB (113). In a very similar study, Sacha et al. depleted CD8 T cells from elite controlling macaques and found robust Gag- and Nef-specific CD4 T cell responses during the re-establishment of viral control (114). Interestingly, the SIV-specific CD4 T cells did not control viral replication in CD4 T cells *ex vivo*; however, they did recognize and eliminate SIV-infected macrophages (114). A recent comparison of monkey species in which SIV infection is either pathogenic or non-pathogenic found higher GzmB expression in CD4 T cells from pathogenic rhesus macaques (pathogenic infection) compared to African green monkeys (non-pathogenic infection) (115). Ayala et al. very recently demonstrated cytolytic activity from an SIV Gag-specific CD4 T cell clone that could control viral replication in other CD4 T cells as well as itself (116).

### Human Immunodeficiency Virus

Activated CD4 T cells are the major targets of the HIV-1, and a proportion of HIV-specific CD4 T cells are infected and lost. Despite this, CD4 T cells with a cytotoxic profile are expanded in HIV-1-infected individuals. Compared to controls, Appay et al. showed that HIV-infected individuals exhibited higher numbers of CD4 T cells expressing perforin, GzmA, and GMP-17/TIA-1 (9). Although these cells were detected early in infection, the highest numbers were found in individuals with chronic infection. The mechanism of cytotoxicity appeared to be primarily perforin mediated (9). Over the years, a number of groups have studied CD4 CTL during different stages of HIV-1 infection. During very early untreated primary HIV-1 infection (PHI; <22 days after the onset of symptoms), Zaunders et al. detected a 10- to 20-fold increase in the proportion of bulk CD4 T cells that were highly activated (CD38+++), proliferating (Ki-67+), and expressed the HIV-1 co-receptor CCR5 as well as GMP-17/TIA-1, perforin, and GzmB (117). Within the same group of PHI individuals, Gag-specific IFN-γ+ CD4 T cells exhibited a similar CD38+++, Ki-67+, Bcl-2 low, CD57−, GMP-17/TIA-1+ phenotype (117). We recently confirmed in a second group of individuals with untreated PHI that CD4 T cells are consistently highly activated and express perforin, GMP-17/TIA-1, GzmB, and GzmK (15). Recently, Johnson et al. characterized HIV-specific CD107a+ CD4 T cells from individuals with PHI using Fluidigm analysis. They found that CD107a+ IFN-γ+ CD4 T cells shared a transcriptional profile with HIV-specific CD8 CTL, including expression of Gzms A, B, K, and perforin; importantly, the HIV-specific CD107a+ CD4 T cells exhibited similar killing activity to HIV-specific CD8 CTL (20).

Importantly, CD4 CTL emerge early during HIV-1 infection, correlate with acute viral load, and are associated with early viral load set point (20). Soghoian et al. performed a longitudinal study of untreated individuals with early PHI and found that individuals who controlled viral replication within 12 months of infection had a significant expansion of HIV-specific CD4 T cells compared to individuals who progressed to higher viral set points (19). Viral controllers had higher expression of the degranulation marker CD107a on Gag-specific CD4 T cells compared to non-controllers, and GzmA+ HIV-specific CD4 T cell responses at baseline were predictive of slower disease progression (19). Together, these studies clearly support a role for CD4 CTL in controlling early viral replication and contributing to delayed disease progression.

CD4 CTL may confer protection from disease progression through several mechanisms. First, p24 and Nef-specific CD4 CTL can suppress HIV replication in both macrophages and T cells *in vitro* (10, 11, 118). In several cases, these CTL responses have been characterized in long-term non-progressors, who have been infected with HIV-1 but have remained asymptomatic for many years. In one such individual, Zaunders et al. identified a very large expansion of circulating CD4 T cells specific for p24, expressing CCR5 and the cytotoxic markers GMP-17/T1A-1, GzmA, and GzmB, that represented 5% of CD4 T cells in this individual (10). No cytotoxic activity was observed *ex vivo*; however, following a 10-day expansion with individual Gag peptides, purified CD4 T cells demonstrated peptide-specific cytotoxicity at even low effector to target ratios (10). Interestingly, these HIV-specific CD4 T cells had originally been identified by their dramatic ability to proliferate *in vitro* in response to p24 (119–121). Second, CD4 CTL may provide immunological pressure on HIV, similar to the well-documented impact of virus-specific CD8 CTL, which can lead to viral escape. Despite the apparent importance of CD4 CTL in the anti-HIV response, a conundrum for many researchers has been why viral escape from CD4 CTL pressure has not been widely identified. Considerable efforts have been made to demonstrate this phenomenon with very few cases noted in the literature. Harcourt et al. described viral variants to p24 and p17 Gag epitopes in the proviral DNA of an HIV+ individual, the epitope variation did not diminish class II binding but were unable to stimulate proliferation of fresh PBMCs or cultured T cell lines (122). More recently, Burwitz et al. studied an elite controlling rhesus macaque that lost viral control and found that post-breakthrough sequencing identified a mutation in Gag p27 targeted by CD4 CTL, indicating the CD4 CTL were able to exert strong immune pressure *in vivo* (123).

### Influenza

As previously discussed, multiple groups have shown that CD4 CTL are generated following acute influenza infection [recently reviewed in Ref. (27, 124)]. Influenza-specific CD4+ CTL were described as early as 1985 by Lukacher et al. (125), and further characterized a decade later when Graham et al. (75) demonstrated protective cytolytic activity in Th1, but not Th2, influenza-specific T cell clones in mice. The precise mechanisms by which CD4 T cells can provide protection against lethal or highly pathogenic influenza infection were identified in a transgenic mouse model of PR8 infection. *In vitro* primed CD4 effector cells adoptively transferred into naïve recipients provided protection in an IFN-γ-independent manner by (A) promoting B cell maturation and antibody production and (B) perforin-mediated cytolytic activity (74). It was further shown that while antibody production was required in the later stages of infection to fully clear the virus, CD4 cytotoxicity was required earlier in infection to control viral replication. Cytolytic activity was Fas/FasL-independent and required the expression of perforin to provide protection, as demonstrated by perforin^−/−^ effectors. Both Brown et al. (72) and McKinstry et al. (73) extended this work, demonstrating that CD4 effectors generated *in vivo* from the lungs of mice given a sublethal PR8 infection could similarly provide protection from lethal infection when transferred to naïve recipient mice. In these studies, CD4 perforin expression not only contributed to recovery and viral control following infection (72, 73) but exerted selective pressure leading to viral escape mutations (73). Those *in vivo*-generated CD4 CTL can provide the same protection from infection as *in vitro*-generated cells provide evidence that the *in vitro* protocols use to induce CD4 cytotoxicity do approximate the mechanisms that lead to naturally occurring CD4 CTL clones.

Influenza-specific CD4 CTL have been identified in human subjects, even in the absence of strain-specific antibodies (26). Preexisting CD4 T cell responses were primarily directed toward the nucleoprotein and matrix protein, which tend to be conserved between strains, and expanded significantly following subsequent viral challenge. Importantly, the frequency of baseline CD4 responses correlated inversely with illness severity following infection, while the expansion of these responses 7 days post-challenge tracked with viral load and illness duration. Further characterization of baseline CD4 responses confirmed the ability of influenza-specific cells to perform perforin/granzyme-mediate killing of B cell targets. Similar CD4 CTL responses are induced in humans following seasonal influenza vaccination (126). Although CD8 CTL vaccine responses are compromised in older adults compared to younger subjects by 10 weeks postvaccination, CD4 cytolytic activity was comparable between all age groups, suggesting that the CD4 CTL response may be more durable in elderly persons. This is consistent with the observation that NKG2D+ CD4 T cells accumulate with age (58), potentially reflecting a preference for CD4 CTL responses in the elderly. Mouse models support this idea, as aged mice exhibited delayed, but higher magnitude, CD4 CTL activity during influenza infection compared to younger mice (127). Additional studies specifically tracking CD4 CTL activity to various infections or vaccines in young and elderly adults will provide further insight into this observation.

### Cytomegalovirus

Infection with human herpesvirus 5, otherwise known as CMV, leads to lifelong asymptomatic infection in healthy hosts. However, in the immunocompromised host such as transplant recipients or untreated HIV-infected individuals, CMV causes serious disease. CMV infects endothelial, epithelial, and glial cells *in vivo*, all of which express MHC class II molecules, particularly following induction by IFN-γ. During primary CMV infection in adults, CMV-specific CD4 T cells have been associated with better clinical outcome (128). These circulating CMV-specific CD4 T cells displayed an effector memory phenotype and produced the Th1 cytokines IFN-γ and TNF, as well as GzmB (59). Following cessation of viral load in primary CMV infection, a population of CD28^−^CD27^−^ CD4 T cells expressing perforin and GzmB have been found to emerge and expand in the circulation of infected individuals (12).

During latent infection, Suni et al. found that CMV-specific CD4 T cells were CD4+CD8^dim^ (8), and Appay et al. showed that these cells were CD28^−^CD27^−^(9) and expressed perforin. Zaunders et al. showed a high proportion of IFN-γ+ CMV-specific CD4 T cells expressed GMP-17/TIA-1; furthermore, a subset of circulating CD4 T cells from CMV-seropositive adults expressed CCR5, GMP-17/TIA-1, GzmA, and CD244, with low expression of GzmB and perforin (10). Purified CD4+ CD8^dim^ T cells possessed higher CMV-specific cytotoxicity compared to their CD4+ CD8^−^ counterparts and were able to lyse whole CMV-loaded EBV-transformed B lymphoblastoid cells *ex vivo* (8). During this latent stage of infection, van Leewen et al. showed that CD4+ CD28− T cells emerged with immediate cytotoxic capacity, that these cells could lyse CMV antigen expressing target cells in a class II-dependent manner, and that CD28− CD4 CTL clones common during latency were rare or absent during early infection (129). Casazza et al. hypothesized that CD4 T cells detected during chronic subclinical CMV infection expressed a specific effector phenotype. They revealed that CMV-specific CD4 T cells expressed IFN-γ, TNF, and MIP-1β in the absence of IL-2, had direct cytolytic activity (CD107a, perforin, GzmA, and B expression), and had a terminally differentiated phenotype (21). They suggested that the lack of IL-2 expression indicates that these CD4 T cells are not present to provide “help” but more likely to have a direct antiviral effect.

Very recently, Pachnio et al. confirmed that CMV-specific CD4 T cells possessed a highly differentiated effector memory phenotype and expressed IFN-γ, TNF, and MIP-1β. Using HLA class II tetramers to characterize CMV-specific CD4 T cells, they found that these cells had an “intense” cytotoxic profile; microarray analysis revealed an upregulation of genes associated with cytotoxic function, such as Gzms A, B, H, granulysin, and perforin, and that these cells also expressed CX3CR1 (a marker of endothelial homing) (130). CMV-specific CD4 CTL showed strong cytotoxic activity *ex vivo* against antigen-loaded targets (130). The same class II tetramer was used by Raeiszadeh et al. to demonstrate CMV-specific CD4 T cell reconstitution following stem cell transplantation; these cells had an effector memory phenotype and contained cytotoxic molecules (131). Shabir et al. followed an unselected group of kidney transplant recipients and examined the CD28− CD4 T cells. These cells were found predominantly in CMV-seropositive patients and expanded posttransplantation; they had an effector memory phenotype and expressed CX3CR1 as well as NKG2D and perforin (132).

### Murine CMV (MCMV)

In MCMV, mice lacking CD4 T cells were shown to have an impaired ability to clear virus from the salivary glands, an important site of viral latency (133). Walton et al. revealed that the mechanism of CD4 T cell immune control in the salivary glands of MCMV infected mice was *via* direct secretion of IFN-γ, which induced antiviral signaling on non-hematopoietic cells (134). Adoptive transfer experiments of MCMV-specific effector CD4 T cells to immune-compromised mice was found to be protective during pathogenic MCMV infection and IFN-γ was a vital mediator of this protective capacity (135). Despite the apparent importance of CD4 T cells in viral control in the above studies, there was no specific link to CD4 CTL. Verma et al. have recently utilized MHC II tetramers to phenotypically and functionally characterize the two first reported I-A^d^-restricted CD4 T cell responses specific for MCMV. They show MCMV-specific CD4 T cells in the liver express higher levels of GzmB than the same cells in the spleen. The organ-dependent expression of GzmB is reflected in *in vivo* CD4 CTL activity, with higher target cell loss in the liver compared to spleen. The data presented in this study suggest that MCMV-specific CD4 T cells can kill target cells in an epitope- and organ-specific manner. Additionally, they show that vaccination with CD4 T cell epitopes leads to reduced viral replication in tissues where CD4 CTL are observed (136).

### Hantavirus

Haantan virus belongs to the *Bunyaviridae* family, and like other viruses in that family, can cause hemorrhagic fever with renal syndrome (HFRS). Screening of CD4 responses with peptide pools derived from the NTNV glycoprotein revealed a subset of virus-specific CD4 cells that expressed GzmB, perforin, and/or CD107a (29). These cells were found at significantly higher frequencies and exhibited greater cytotoxicity among patients with mild/moderate disease compared to those with severe or critical illness. Furthermore, the frequency of GzmB+ CD4 cells correlated inversely with RNA load during acute HFRS, suggesting a potential impact of CD4 CTL responses in the control of infection.

## Infections That Evade CD4 CTL Responses

### Epstein–Barr Virus

Epstein–Barr virus is a ubiquitous herpesvirus that infects 95% of the human population by adulthood (137), taking the form of infectious mononucleosis in the acute phase. The high global prevalence of EBV infection results in EBV accounting for a 1% worldwide cancer incidence rate, with Hodgkin lymphoma, non-Hodgkin lymphoma, nasopharyngeal carcinoma, and gastric cancers being the most common EBV-associated malignancies (138). The identification of cytotoxic EBV-specific CD4 responses dates back to 1984 (139), and similar responses have been identified in the mouse model of infection with γ-herpesvirus 68, in which cytolytic killing of virally infected cells by CD4 T cells has been observed *in vivo* (140, 141). Much of the work in this field has focused on identifying EBV epitopes from various stages of the lytic and latent phases of infection that may overcome viral immune evasion. Multiple studies have described CD4 CTL responses against latent-cycle antigens that evade recognition by CD8 CTL (142–144). The EBNA1 protein is expressed in all forms of EBV-related malignancy, making it an ideal candidate for immunotherapy; efforts to target a CD8 CTL response against the protein have been challenging, however, as a glycine-alanine repeat within the protein prevents presentation by MHC class I (145). Several groups have since shown that CD4 CTL can recognize EBNA1 epitopes and kill infected B cells (76, 142, 144). Interestingly, these CD4 CTL clones exhibited distinct phenotypic and functional characteristics compared to the influenza-specific CD4 CTL described previously; they exhibited a Th0 phenotype (with secretion of both IFN-γ and IL-4) and killed infected cells *via* Fas/FasL interactions rather than perforin secretion (144).

The relevance of these responses *in vivo* and in immunotherapy approaches, however, is called into question with the observation that EBNA1-specific CD4 CTL identified *in vitro* only poorly recognize native lymphoblastoid cell lines (LCLs) expressing EBNA1. Although this is commonly attributed to the protein’s limited access to the MHC class II presentation pathway (146), a recent study identifies EBV miRNA expression as a novel immune evasion mechanism (147). EBV-derived miRNAs suppress Th1 differentiation pathways, interfere with MHC class II antigen presentation, and subsequently inhibit the activation of EBV-specific CD4 CTL. Encouragingly, Long et al. (143) recently demonstrated that CD4 CTL responses to a variety of lytic cycle antigens could efficiently recognize EBV-transformed LCLs containing only a small proportion of lytically infected cells. This CD4 response was possible due to the uptake of lytic antigens by latently infected cells, which then efficiently processed and displayed epitopes for CD4 recognition. CD4 CTL also responded to a substantially broader array of lytic cycle proteins across the immediate early, early, and late phases compared to CD8 CTL, which skew toward immediate early proteins. Several other studies have identified cytolytic CD4 responses to epitopes in the lytic protein BHRF1 (in HLA-DR*0401-positive subjects) (24), as well as BLLF1, BALF4, and BZLF1 (148). *In vitro*, T cell recognition of BLLF1/BALF4/BZLF1 epitopes did not require productive infection, could occur in the context of viral transfer to bystander B cells and was capable of controlling proliferation of infected B cells *via* cytolysis (148), further confirming the therapeutic potential of CD4 CTL (149).

Therapeutic induction of effector CD4 T cell responses against EBV antigen to fight malignancies demonstrate promise in animal models; effective killing of virus-induced tumor cells following treatment with a CD137 agonist was seen in CD8 T cell-deficient mice (150). In the same murine model, CD137 signaling improved expression of pro-inflammatory cytokines and cytotoxic effector molecules in tumor-specific CD4 T cells, coincident with upregulation of Eomes and Tbox (151); these cells were able to kill virus-induced tumor cells *in vivo*. Moving in the preclinical direction of immunotherapy, EBNA1-specific CD4 T cells expressing effector cytokines and GzmB were induced following therapeutic EBV vaccination against rhEBNA1 fused to the herpes simplex virus glycoprotein D, in rhesus macaques infected with lymphocryptovirus (152). Phase I human trials focus upon nasopharyngeal carcinoma, a malignancy more prevalent in South East Asia (153). Recombinant vaccinia virus encoding EBNA1 and LMP2 was applied intradermally to boost immunity in EBV-associated nasopharyngeal carcinoma patients. T-cell responses were detected postvaccination by IFN-γ ELISpot and mapped to MHC class II epitopes for both vaccine-encoded proteins (154). Constructs specifically designed to target CD4 T cell response to EBV by encoding the CD4 T cell epitope enriched C-terminal EBNA1-fused to LMP2 (153), demonstrated functional differentiation and *in vitro* targeting of antigen-loaded cells, though epitope mapping was not performed. Efficacy studies thus demonstrate generation of CD4 CTL, which target antigen constitutively expressed in EBV-associated cancer, giving promise to development of Phase II trials and complementary therapies that lead to remission.

## Infections in Which CD4 CTL may be Pathogenic

### Dengue Virus

The fact that DENV infection elicits cytotoxic CD4 T cell responses in humans has been known since 1989 (30), whether these cells are protective or pathogenic is currently unclear. Secondary infection with a dengue serotype different from the primary infection can lead to dengue hemorrhagic fever (DHF) and dengue shock syndrome (DSS). Multiple early studies demonstrated that DENV-specific CD4 CTL are cross-reactive against multiple serotypes (30–33), leading to the hypothesis that CD4 cytotoxicity may contribute to DHF and DSS. Additionally, Gagnon et al. (77) reported that capsid protein-specific CD4 CTL could mediate killing of both APCs (*via* perforin expression) and HepG2 liver cells (*via* Fas/FasL recognition). The authors concluded that the killing of liver cells may also implicate CD4 CTL in the manifestation of liver disease that is frequently observed upon secondary infection. More recently, however, Weiskopf et al. performed a more comprehensive characterization of *ex vivo* DENV-specific CD4 T cells and confirmed that these cells are strongly biased toward a cytolytic phenotype (155). Repeated dengue infection was associated with an expansion of DENV-specific CCR7−CD45RA+ CD4 memory cells that expressed CD8α, CD107a, perforin, granzyme, Eomes, and CX3CR1 and were capable of killing target cells. In contrast to previous studies, Weiskopf suggested that CD4 CTL may play a role in reducing the severity of dengue infection due to the observation that the frequency of these cells is greater among individuals carrying the protective HLA-DRB1*04 allele. It is unclear whether any of the individuals with multiple dengue infections in this study experienced DHF/DSS; comparisons of CD4 CTL activity not only among individuals with or without protective HLA alleles but also among those with or without severe complications of secondary infection will be highly informative in the future.

### Viral Hepatitis

Relatively little is known about CD4 CTL responses in viral hepatitis. A comparison of individuals infected with Hepatitis B virus (HBV), HCV, and HBV/Hepatitis D virus (HDV) co-infection revealed a variable, but significantly increased, proportion of CD4 T cells expressing perforin *ex vivo* compared to healthy controls; in some individuals, up to 25% of bulk CD4 T cells were perforin+ (28). Perforin levels were significantly higher in HDV-infected subjects compared to HBV or HCV monoinfection and were, in fact, comparable to HIV-infected patients. As previously mentioned, the fact that liver hepatocytes may be a target of chronically induced CD4 CTL raises the issue of whether these cells contribute to immunopathology. Among all hepatitis patients, CD4 perforin expression correlated with aspartate aminotransferase levels and platelet counts and was highest among patients with the most advanced disease. Additional work, including longitudinal studies, will be required to better define the role of CD4 CTL in viral hepatitis, as well as the antigen specificity of these CTL. While one study describes an HCV-specific TCR that can confer cytolytic activity when transduced into CD4 T cells (156), the *in vivo* CD4 CTL repertoire generated during chronic hepatitis infection remains to be described.

## Other Viral Infections

### Poxviruses—Ectromelia and Vaccinia Viruses

CD4 CTL play an important role in the response to ectromelia virus, the pathogen responsible for mousepox. During acute ectromelia virus infection, a large number of CD4 T cells that express GzmB were found in the draining lymph nodes and liver of infected mice (157). These CD4 CTL demonstrated *in vivo* MHC II-restricted CTL activity that was perforin dependent. Defective control of ectromelia virus was found in mice with a specific deficiency of perforin in their CD4 T cells (157). Comparisons of ectromelia virus and vaccinia virus (the active constituent of the smallpox vaccine) infection in mice showed that ectromelia virus induced significantly more CD4 CTL than vaccinia with different epitope-specific CD4 cells exhibiting different cytotoxic frequencies (158). These results reveal that not all viral infections result in the generation of CD4 CTL with the same cytolytic ability.

The first descriptions of CD4 CTL in response to vaccinia virus in humans were from Littaua et al. and Demkowicz et al. (159, 160) who established CD3+CD4+CD8−Leu11− cytotoxic T cell lines and clones from vaccinated individuals. These clones and cell lines were vaccinia virus-specific and lysed target cells in an HLA class II-restricted manner. The mechanism of lysis was not established for these CD4 cell lines or clones; however, further studies of vaccinia virus-specific cytotoxic CD4 T cell lines have shown that lysis was inhibited by concanamycin A, an inhibitor of perforin, but not by an anti-Fas antibody (161). Zaunders et al. (14) have shown that activated CD4 T cells detected in the early primary immune response to immunization with vaccinia virus expressed the cytotoxic granule marker TIA-1. The IFN-γ+ vaccinia virus-specific CD4 T cells detected in this study appeared as early as 11 days postvaccination and were shown to be predominantly TIA-1+ and were CD57−. A more recent study of the genetic profile of these activated CD4 T cells in the acute response to vaccinia virus has shown that these cells have a prominent CTL profile with upregulation of CTL associated genes including GzmK GzmA, KLRB1/CD161, Rab27a, and granulysin (15). The genes for perforin and TIA-1 were also upregulated; interestingly, the expression of *GzmB* was downregulated. From the same study, vaccinia virus-specific CD4 T cell lines were generated from a vaccinated individual at 13 days and 12 months postvaccination. These lines were shown to upregulate the machinery of cytotoxic degranulation and subsequently lyse HLA-matched target cells, loaded with autologous vaccinia virus peptides (15).

### Parvovirus

Human parvovirus B19 is a relatively common viral infection; although it is often asymptomatic, infection has been associated with aggravation of rheumatoid arthritis and systemic lupus erythematosus (162). CD4 cells from B19 seropositive individuals exhibit perforin and GzmB expression following antigenic stimulation and are cytotoxic, albeit following a relatively long 5-day stimulation (34). Interestingly, CD4 CTL in seropositive donors co-expressed IL-17 and CD56, two markers not previously associated with cytotoxic CD4 responses in other infections. The importance of these markers in CD4 CTL function and immunity to parvovirus B19 remains to be explored.

## Vaccine-Induced CD4 CTL

The mounting evidence that CD4 CTL are an important component of protective immunity against many infectious diseases suggests that eliciting such cytotoxic responses may boost vaccine efficacy against infections such as HIV and influenza. Indeed, Watson et al. (163) recently showed that the highly effective live-attenuated yellow fever vaccine elicits both CD8 and CD4 CTL responses in a mouse model of infection. Both immune sera and CD4 T cells were required for full protection against fatal infection, which closely mimics the requirements described by Brown et al. (74) for protection from fatal influenza infection and provides support for the goal of vaccine-elicited CD4 CTL responses.

Based on the evidence suggesting that the induction of Th1 responses at the site of infection in the context of IL-2 and an inflammatory signal should induce an optimal CD4 CTL response against influenza infection, Vogel and Brown (164), tested the efficacy of intranasal immunization of inactivated influenza with CpG adjuvant. This vaccine induced high levels of inflammatory and chemotactic transcripts and significantly reduced viral burden in response to heterologous challenge. Compared to vaccination with inactivated influenza alone, the addition of CpG induced significantly higher GzmB expression in lung CD4 T cells, which persisted after challenge with lethal PR8 infection. This approach may therefore be a good candidate for generating adaptive memory responses at the site of infection that are capable of limiting the replication of heterotypic viruses, particularly if well-conserved epitopes were chosen for vaccination. To that end, Babon et al. (165) identified a CD4 T cell epitope in the fusion peptide of the HA that is conserved across all influenza A HA subtypes as well as the influenza B HA protein. A CD4 T cell clone recognizing this epitope exhibits cytotoxicity to a variety of influenza strains, including avian H5N1 virus. Importantly, the CD4 clone is likely restricted by a common HLA allele, making this epitope an intriguing candidate for future vaccine studies. Using TLR-stimulating adjuvants to boost flu-specific CTL responses to conserved epitopes represents a novel avenue for future vaccine candidates (166).

The induction of CD4 CTL responses by an HIV vaccine may be similarly advantageous. Terahara et al. studied vaccine-induced SIV-specific CD4 T cell responses and found that CD107a+ vaccine-elicited CD4 T cells were more resistant to SIV infection than CD107a- cells, suggesting that CD4 CTL may be an ideal response to elicit during vaccination in order to avoid generating increased levels of target cells (167). While evidence does suggest the protective role of CD4 CTL induced early after infection, less work has focused on determining the efficacy of vaccine-induced CD4 CTL in humans. Vaccine-induced cell-mediated responses elicited by the modestly protective RV144 vaccine were assessed in HIV-uninfected individuals, and Env-specific polyfunctional effector memory CD4 T cells were detected along with high expression of CD107a in proliferating HIV-specific CD4 T cells (17). HIV-specific CD4 T cell lines grown from HIV-uninfected vaccinees were found to be polyfunctional and have cytolytic capacity in response to an Env peptide pool, suggesting that the vaccine did elicit some cytotoxic CD4 responses (17). Although not specifically linked to CD4 CTL, it is of interest to note that recent reanalysis of data from the original RV144 correlate analysis (168) has shown that two vaccine-induced polyfunctional CD4 T cell subsets are associated with decreased risk of HIV infection (169). A more specific analysis of CD4 CTL activity in infected and uninfected vaccine recipients is warranted in future trials.

## Conclusion/Future Directions

Major histocompatibility complex class II-restricted CD4 T cells with a cytotoxic phenotype are a prominent component of the antiviral immune response. These CD4 CTL have been identified in response to a plethora of viral infections affecting mice, non-human primates, and humans, and many aspects of their role in immunity remain unanswered. Despite the apparent importance of cytotoxic CD4 T cells in the immune response to many viruses, there is to date a paucity of reports in the literature of these cells placing any immune pressure on the virus as evidenced by CD4 CTL escape. The lack of these data is intriguing and should be further studied to understand the immune pressure exerted by the antiviral CD4 CTL response. With regard to T regulatory cells, the role of granzymes in regulatory function needs to be confirmed, and differences between studies in humans and mice require clarification.

Future studies of CD4 CTL should concentrate on single-cell transcriptomics to further understand and define the CD4 CTL lineage. A large body of studies have been conducted on murine CD4 CTL; however, it is important to understand if such studies provide equivalent and translational information about human CD4 CTL. Further studies on how best to generate antigen-specific CD4 CTL *in vitro* for cell therapy and immunization are also warranted.

## Author Contributions

JJ, DB, SK, AK, JZ, and CM organized, wrote, and edited the manuscript.

## Conflict of Interest Statement

The authors declare that the research was conducted in the absence of any commercial or financial relationships that could be construed as a potential conflict of interest.

## References

[1] Billings P Burakoff S Dorf M Benacerraf B. Cytotoxic T lymphocytes specific for I region determinants do not require interactions with H-2K or D gene products. J Exp Med (1977) 145:1387–92. 10.1084/jem.145.5.1387 67179 PMC2180671

[2] Dennert G Swain SL Waterfield JD Warner JF Dutton RW. Fine specificity mapping of two allospecific T cell lines: recognition of private specificities in the H-2 IA subregion. Eur J Immunol (1981) 11:62–4. 10.1002/eji.1830110113 6163637

[3] Swain SL Dennert G Wormsley S Dutton RW. The Lyt phenotype of a long-term allospecific T cell line. Both helper and killer activities to IA are mediated by Ly-1 cells. Eur J Immunol (1981) 11:175–80. 10.1002/eji.1830110304 6972304

[4] Krensky A Reiss C Mier J Strominger J Burakoff S. Long-term human cytolytic T-cell lines allospecific for HLA-DR6 antigen are OKT4+. Proc Natl Acad Sci U S A (1982) 79:2365–9. 10.1073/pnas.79.7.2365 6980419 PMC346194

[5] Maimone M Morrison L Braciale V Braciale T. Features of target cell lysis by class I and class II MHC-restricted cytolytic T lymphocytes. J Immunol (1986) 137:3639–43. 3491143

[6] Fleischer B. Acquisition of specific cytotoxic activity by human T4+ T lymphocytes in culture. Nature (1984) 308:365–7. 10.1038/308365a0 6608693

[7] Lindsley MD Torpey DJ III Rinaldo CR Jr. HLA-DR-restricted cytotoxicity of cytomegalovirus-infected monocytes mediated by Leu-3-positive T cells. J Immunol (1986) 136:3045–51. 2420881

[8] Suni M Ghanekar S Houck D Maecker H Wormsley S Picker L CD4(+)CD8(dim) T lymphocytes exhibit enhanced cytokine expression, proliferation and cytotoxic activity in response to HCMV and HIV-1 antigens. Eur J Immunol (2001) 31:2512–20. 10.1002/1521-4141(200108)31:8<2512::AID-IMMU2512>3.0.CO;2-M 11500836

[9] Appay V Zaunders JJ Papagno L Sutton J Jaramillo A Waters A Characterization of CD4(+) CTLs ex vivo. J Immunol (2002) 168:5954–8. 10.4049/jimmunol.168.11.5954 12023402

[10] Zaunders JJ Dyer WB Wang B Munier ML Miranda-Saksena M Newton R Identification of circulating antigen-specific CD4+ T lymphocytes with a CCR5+, cytotoxic phenotype in an HIV-1 long-term nonprogressor and in CMV infection. Blood (2004) 103:2238–47. 10.1182/blood-2003-08-2765 14645006

[11] Norris P Moffett H Yang O Kaufmann D Clark M Addo M Beyond help: direct effector functions of human immunodeficiency virus type 1-specific CD4(+) T cells. J Virol (2004) 78:8844–51. 10.1128/JVI.78.16.8844-8851.2004 15280492 PMC479080

[12] van Leeuwen E Remmerswaal E Vossen M Rowshani A Wertheim-van Dillen P van Lier R Emergence of a CD4+CD28- granzyme B+, cytomegalovirus-specific T cell subset after recovery of primary cytomegalovirus infection. J Immunol (2004) 173:1834–41. 10.4049/jimmunol.173.3.1834 15265915

[13] Brown D. Cytolytic CD4 cells: direct mediators in infectious disease and malignancy. Cell Immunol (2010) 262:89–95. 10.1016/j.cellimm.2010.02.008 20236628 PMC2874968

[14] Zaunders JJ Dyer WB Munier ML Ip S Liu J Amyes E CD127+CCR5+CD38+++ CD4+ Th1 effector cells are an early component of the primary immune response to vaccinia virus and precede development of interleukin-2+ memory CD4+ T cells. J Virol (2006) 80:10151–61. 10.1128/JVI.02670-05 17005692 PMC1617315

[15] Munier CM van Bockel D Bailey M Ip S Xu Y Alcantara S The primary immune response to vaccinia virus vaccination includes cells with a distinct cytotoxic effector CD4 T-cell phenotype. Vaccine (2016) 34:5251–61. 10.1016/j.vaccine.2016.09.009 27639281

[16] Wahid R Cannon M Chow M. Virus-specific CD4+ and CD8+ cytotoxic T-cell responses and long-term T-cell memory in individuals vaccinated against polio. J Virol (2005) 79:5988–95. 10.1128/JVI.79.10.5988-5995.2005 15857985 PMC1091702

[17] de Souza M Ratto-Kim S Chuenarom W Schuetz A Chantakulkij S Nuntapinit B The Thai phase III trial (RV144) vaccine regimen induces T cell responses that preferentially target epitopes within the V2 region of HIV-1 envelope. J Immunol (2012) 188:5166–76. 10.4049/jimmunol.1102756 22529301 PMC3383859

[18] Nemes E Bertoncelli L Lugli E Pinti M Nasi M Manzini L Cytotoxic granule release dominates gag-specific CD4+ T-cell response in different phases of HIV infection. AIDS (2010) 24:947–57. 10.1097/QAD.0b013e328337b144 20179574

[19] Soghoian D Jessen H Flanders M Sierra-Davidson K Cutler S Pertel T HIV-specific cytolytic CD4 T cell responses during acute HIV infection predict disease outcome. Sci Transl Med (2012) 4:123ra25. 10.1126/scitranslmed.3003165 22378925 PMC3918726

[20] Johnson S Eller M Teigler JE Maloveste SM Schultz BT Soghoian DZ Cooperativity of HIV-specific cytolytic CD4 T cells and CD8 T cells in control of HIV viremia. J Virol (2015) 89:7494–505. 10.1128/JVI.00438-15 25972560 PMC4505667

[21] Casazza JP Betts MR Price DA Precopio ML Ruff LE Brenchley JM Acquisition of direct antiviral effector functions by CMV-specific CD4+ T lymphocytes with cellular maturation. J Exp Med (2006) 203:2865–77. 10.1084/jem.20052246 17158960 PMC2118179

[22] Saez-Borderias A Guma M Angulo A Bellosillo B Pende D Lopez-Botet M. Expression and function of NKG2D in CD4+ T cells specific for human cytomegalovirus. Eur J Immunol (2006) 36:3198–206. 10.1002/eji.200636682 17109473

[23] Omiya R Buteau C Kobayashi H Paya C Celis E. Inhibition of EBV-induced lymphoproliferation by CD4(+) T cells specific for an MHC class II promiscuous epitope. J Immunol (2002) 169:2172–9. 10.4049/jimmunol.169.4.2172 12165547

[24] Landais E Saulquin X Scotet E Trautmann L Peyrat M Yates J Direct killing of Epstein-Barr virus (EBV)-infected B cells by CD4 T cells directed against the EBV lytic protein BHRF1. Blood (2004) 103:1408–16. 10.1182/blood-2003-03-0930 14563644

[25] Haigh T Lin X Jia H Hui E Chan A Rickinson A EBV latent membrane proteins (LMPs) 1 and 2 as immunotherapeutic targets: LMP-specific CD4+ cytotoxic T cell recognition of EBV-transformed B cell lines. J Immunol (2008) 180:1643–54. 10.4049/jimmunol.180.3.1643 18209060

[26] Wilkinson T Li C Chui C Huang A Perkins M Liebner J Preexisting influenza-specific CD4+ T cells correlate with disease protection against influenza challenge in humans. Nat Med (2012) 18:274–80. 10.1038/nm.2612 22286307

[27] Brown DM Lampe AT Workman AM. The differentiation and protective function of cytolytic CD4 T cells in influenza infection. Front Immunol (2016) 7:93. 10.3389/fimmu.2016.00093 27014272 PMC4783394

[28] Aslan N Yurdaydin C Wiegand J Greten T Ciner A Meyer M Cytotoxic CD4 T cells in viral hepatitis. J Viral Hepat (2006) 13:505–14. 10.1111/j.1365-2893.2006.00723.x 16901280

[29] Ma Y Yuan B Zhuang R Zhang Y Liu B Zhang C Hantaan virus infection induces both Th1 and Th granzyme B+ cell immune responses that associated with viral control and clinical outcome in humans. PLoS Pathog (2015) 11:e1004788. 10.1371/journal.ppat.1004788 25836633 PMC4383613

[30] Kurane I Meager A Ennis FA Dengue virus-specific human T cell clones. Serotype crossreactive proliferation, interferon gamma production, and cytotoxic activity. J Exp Med (1989) 170:763–75. 10.1084/jem.170.3.763 2475573 PMC2189437

[31] Gagnon SJ Zeng W Kurane I Ennis FA. Identification of two epitopes on the dengue 4 virus capsid protein recognized by a serotype-specific and a panel of serotype-cross-reactive human CD4+ cytotoxic T-lymphocyte clones. J Virol (1996) 70:141–7. 8523518 10.1128/jvi.70.1.141-147.1996 PMC189798

[32] Kurane I Brinton MA Samson AL Ennis FA. Dengue virus-specific, human CD4+ CD8- cytotoxic T-cell clones: multiple patterns of virus cross-reactivity recognized by NS3-specific T-cell clones. J Virol (1991) 65:1823–8. 1705990 10.1128/jvi.65.4.1823-1828.1991 PMC239991

[33] Zeng L Kurane I Okamoto Y Ennis FA Brinton MA. Identification of amino acids involved in recognition by dengue virus NS3-specific, HLA-DR15-restricted cytotoxic CD4+ T-cell clones. J Virol (1996) 70:3108–17. 8627790 10.1128/jvi.70.5.3108-3117.1996 PMC190173

[34] Kumar A Perdomo MF Kantele A Hedman L Hedman K Franssila R. Granzyme B mediated function of parvovirus B19-specific CD4(+) T cells. Clin Transl Immunology (2015) 4:e39. 10.1038/cti.2015.13 26246896 PMC4524951

[35] Kischkel F Hellbardt S Behrmann I Germer M Pawlita M Krammer P Cytotoxicity-dependent APO-1 (Fas/CD95)-associated proteins form a death-inducing signaling complex (DISC) with the receptor. EMBO J (1995) 14:5579–88. 8521815 10.1002/j.1460-2075.1995.tb00245.x PMC394672

[36] Nagata S Golstein P. The Fas death factor. Science (1995) 267:1449–56. 10.1126/science.7533326 7533326

[37] Krammer P. CD95’s deadly mission in the immune system. Nature (2000) 407:789–95. 10.1038/35037728 11048730

[38] Trapani J Smyth M. Functional significance of the perforin/granzyme cell death pathway. Nat Rev Immunol (2002) 2:735–47. 10.1038/nri911 12360212

[39] Peters PJ Borst J Oorschot V Fukuda M Krahenbuhl O Tschopp J Cytotoxic T lymphocyte granules are secretory lysosomes, containing both perforin and granzymes. J Exp Med (1991) 173:1099–109. 10.1084/jem.173.5.1099 2022921 PMC2118839

[40] Bossi G Griffiths GM. CTL secretory lysosomes: biogenesis and secretion of a harmful organelle. Semin Immunol (2005) 17:87–94. 10.1016/j.smim.2004.09.007 15582491

[41] Krzewski K Cullinane AR. Evidence for defective Rab GTPase-dependent cargo traffic in immune disorders. Exp Cell Res (2013) 319:2360–7. 10.1016/j.yexcr.2013.06.012 23810987 PMC3759575

[42] Masson D Tschopp J. Isolation of a lytic, pore-forming protein (perforin) from cytolytic T-lymphocytes. J Biol Chem (1985) 260:9069–72. 3874868

[43] Podack E Young J Cohn Z. Isolation and biochemical and functional characterization of perforin 1 from cytolytic T-cell granules. Proc Natl Acad Sci U S A (1985) 82:8629–33. 10.1073/pnas.82.24.8629 2417226 PMC390971

[44] Chowdhury D Lieberman J. Death by a thousand cuts: granzyme pathways of programmed cell death. Annu Rev Immunol (2008) 26:389–420. 10.1146/annurev.immunol.26.021607.090404 18304003 PMC2790083

[45] Cullen S Brunet M Martin S. Granzymes in cancer and immunity. Cell Death Differ (2010) 17:616–23. 10.1038/cdd.2009.206 20075940

[46] Anthony D Andrews D Watt S Trapani J Smyth M. Functional dissection of the granzyme family: cell death and inflammation. Immunol Rev (2010) 235:73–92. 10.1111/j.0105-2896.2010.00907.x 20536556

[47] Bovenschen N Kummer J. Orphan granzymes find a home. Immunol Rev (2010) 235:117–27. 10.1111/j.0105-2896.2010.00889.x 20536559

[48] Smyth M Kelly J Sutton V Davis J Browne K Sayers T Unlocking the secrets of cytotoxic granule proteins. J Leukoc Biol (2001) 70:18–29. 11435481

[49] Barry M Bleackley R. Cytotoxic T lymphocytes: all roads lead to death. Nat Rev Immunol (2002) 2:401–9. 10.1038/nri819 12093006

[50] Grossman W Revell P Lu Z Johnson H Bredemeyer A Ley T. The orphan granzymes of humans and mice. Curr Opin Immunol (2003) 15:544–52. 10.1016/S0952-7915(03)00099-2 14499263

[51] Bovenschen N Quadir R van den Berg A Brenkman A Vandenberghe I Devreese B Granzyme K displays highly restricted substrate specificity that only partially overlaps with granzyme A. J Biol Chem (2009) 284:3504–12. 10.1074/jbc.M806716200 19059912

[52] Hildemann S Eberlein J Davenport B Nguyen T Victorino F Homann D. High efficiency of antiviral CD4(+) killer T cells. PLoS One (2013) 8:e60420. 10.1371/journal.pone.0060420 23565245 PMC3614903

[53] Lin L Couturier J Yu X Medina MA Kozinetz CA Lewis DE. Granzyme B secretion by human memory CD4 T cells is less strictly regulated compared to memory CD8 T cells. BMC Immunol (2014) 15:36. 10.1186/s12865-014-0036-1 25245659 PMC4195902

[54] Anderson P Nagler-Anderson C O’Brien C Levine H Watkins S Slayter HS A monoclonal antibody reactive with a 15-kDa cytoplasmic granule-associated protein defines a subpopulation of CD8+ T lymphocytes. J Immunol (1990) 144:574–82. 2104899

[55] Brenchley JM Karandikar NJ Betts MR Ambrozak DR Hill BJ Crotty LE Expression of CD57 defines replicative senescence and antigen-induced apoptotic death of CD8+ T cells. Blood (2003) 101:2711–20. 10.1182/blood-2002-07-2103 12433688

[56] Appay V The physiological role of cytotoxic CD4(+) T-cells: the holy grail? Clin Exp Immunol (2004) 138:10–3. 10.1111/j.1365-2249.2004.02605.x 15373899 PMC1809194

[57] Schreeder DM Pan J Li FJ Vivier E Davis RS. FCRL6 distinguishes mature cytotoxic lymphocytes and is upregulated in patients with B-cell chronic lymphocytic leukemia. Eur J Immunol (2008) 38:3159–66. 10.1002/eji.200838516 18991291 PMC2742621

[58] Alonso-Arias R Moro-Garcia MA Lopez-Vazquez A Rodrigo L Baltar J Garcia FM NKG2D expression in CD4+ T lymphocytes as a marker of senescence in the aged immune system. Age (Dordr) (2011) 33:591–605. 10.1007/s11357-010-9200-6 21210234 PMC3220398

[59] Gamadia LE Rentenaar RJ van Lier RA ten Berge IJ Properties of CD4(+) T cells in human cytomegalovirus infection. Hum Immunol (2004) 65:486–92. 10.1016/j.humimm.2004.02.020 15172448

[60] van de Berg PJ van Leeuwen EM ten Berge IJ van Lier R. Cytotoxic human CD4(+) T cells. Curr Opin Immunol (2008) 20:339–43. 10.1016/j.coi.2008.03.007 18440213

[61] Hirahara K Nakayama T. CD4+ T-cell subsets in inflammatory diseases: beyond the Th1/Th2 paradigm. Int Immunol (2016) 28:163–71. 10.1093/intimm/dxw006 26874355 PMC4889886

[62] Pearce E Mullen A Martins G Krawczyk C Hutchins A Zediak V Control of effector CD8+ T cell function by the transcription factor eomesodermin. Science (2003) 302:1041–3. 10.1126/science.1090148 14605368

[63] Intlekofer A Banerjee A Takemoto N Gordon S Dejong C Shin H Anomalous type 17 response to viral infection by CD8+ T cells lacking T-bet and eomesodermin. Science (2008) 321:408–11. 10.1126/science.1159806 18635804 PMC2807624

[64] Hua L Yao S Pham D Jiang L Wright J Sawant D Cytokine-dependent induction of CD4+ T cells with cytotoxic potential during influenza virus infection. J Virol (2013) 87:11884–93. 10.1128/JVI.01461-13 23986597 PMC3807312

[65] Glimcher L Townsend M Sullivan B Lord G. Recent developments in the transcriptional regulation of cytolytic effector cells. Nat Rev Immunol (2004) 4:900–11. 10.1038/nri1490 15516969

[66] Qui HZ Hagymasi AT Bandyopadhyay S St Rose MC Ramanarasimhaiah R Menoret A CD134 plus CD137 dual costimulation induces eomesodermin in CD4 T cells to program cytotoxic Th1 differentiation. J Immunol (2011) 187:3555–64. 10.4049/jimmunol.1101244 21880986 PMC3178659

[67] Eshima K Chiba S Suzuki H Kokubo K Kobayashi H Iizuka M Ectopic expression of a T-box transcription factor, eomesodermin, renders CD4(+) Th cells cytotoxic by activating both perforin- and FasL-pathways. Immunol Lett (2012) 144:7–15. 10.1016/j.imlet.2012.02.013 22425747

[68] Takeuchi A Badr Mel S Miyauchi K Ishihara C Onishi R Guo Z CRTAM determines the CD4+ cytotoxic T lymphocyte lineage. J Exp Med (2016) 213:123–38. 10.1084/jem.20150519 26694968 PMC4710199

[69] Mucida D Husain MM Muroi S van Wijk F Shinnakasu R Naoe Y Transcriptional reprogramming of mature CD4(+) helper T cells generates distinct MHC class II-restricted cytotoxic T lymphocytes. Nat Immunol (2013) 14:281–9. 10.1038/ni.2523 23334788 PMC3581083

[70] Reis BS Rogoz A Costa-Pinto FA Taniuchi I Mucida D Mutual expression of the transcription factors Runx3 and ThPOK regulates intestinal CD4(+) T cell immunity. Nat Immunol (2013) 14:271–80. 10.1038/ni.2518 23334789 PMC3804366

[71] Reis BS Hoytema van Konijnenburg DP Grivennikov SI Mucida D. Transcription factor T-bet regulates intraepithelial lymphocyte functional maturation. Immunity (2014) 41:244–56. 10.1016/j.immuni.2014.06.017 25148025 PMC4287410

[72] Brown DM Lee S Garcia-Hernandez Mde L Swain SL. Multifunctional CD4 cells expressing gamma interferon and perforin mediate protection against lethal influenza virus infection. J Virol (2012) 86:6792–803. 10.1128/JVI.07172-11 22491469 PMC3393557

[73] McKinstry K Strutt T Kuang Y Brown D Sell S Dutton R Memory CD4+ T cells protect against influenza through multiple synergizing mechanisms. J Clin Invest (2012) 122:2847–56. 10.1172/JCI63689 22820287 PMC3408751

[74] Brown D Dilzer A Meents D Swain S. CD4 T cell-mediated protection from lethal influenza: perforin and antibody-mediated mechanisms give a one-two punch. J Immunol (2006) 177:2888–98. 10.4049/jimmunol.177.5.2888 16920924

[75] Graham MB Braciale VL Braciale TJ. Influenza virus-specific CD4+ T helper type 2 T lymphocytes do not promote recovery from experimental virus infection. J Exp Med (1994) 180:1273–82. 10.1084/jem.180.4.1273 7931062 PMC2191682

[76] Paludan C Bickham K Nikiforow S Tsang ML Goodman K Hanekom WA Epstein-Barr nuclear antigen 1-specific CD4(+) Th1 cells kill Burkitt’s lymphoma cells. J Immunol (2002) 169:1593–603. 10.4049/jimmunol.169.3.1593 12133989

[77] Gagnon SJ Ennis FA Rothman AL. Bystander target cell lysis and cytokine production by dengue virus-specific human CD4(+) cytotoxic T-lymphocyte clones. J Virol (1999) 73:3623–9. 10196254 10.1128/jvi.73.5.3623-3629.1999 PMC104137

[78] Brown D Kamperschroer C Dilzer A Roberts D Swain S. IL-2 and antigen dose differentially regulate perforin- and FasL-mediated cytolytic activity in antigen specific CD4+ T cells. Cell Immunol (2009) 257:69–79. 10.1016/j.cellimm.2009.03.002 19338979 PMC2683476

[79] Workman AM Jacobs AK Vogel AJ Condon S Brown DM. Inflammation enhances IL-2 driven differentiation of cytolytic CD4 T cells. PLoS One (2014) 9:e89010. 10.1371/journal.pone.0089010 24586481 PMC3930678

[80] Zheng CF Jones GJ Shi M Wiseman JC Marr KJ Berenger BM Late expression of granulysin by microbicidal CD4+ T cells requires PI3K- and STAT5-dependent expression of IL-2Rbeta that is defective in HIV-infected patients. J Immunol (2008) 180:7221–9. 10.4049/jimmunol.180.11.7221 18490721 PMC2661617

[81] Zheng CF Ma LL Jones GJ Gill MJ Krensky AM Kubes P Cytotoxic CD4(+) T cells use granulysin to kill *Cryptococcus neoformans*, and activation of this pathway is defective in HIV patients. Blood (2007) 109:2049–57. 10.1182/blood-2006-03-009720 17038537

[82] Moore TC Vogel AJ Petro TM Brown DM. IRF3 deficiency impacts granzyme B expression and maintenance of memory T cell function in response to viral infection. Microbes Infect (2015) 17:426–39. 10.1016/j.micinf.2015.03.001 25777301 PMC4479197

[83] Carlier VA VanderElst L Janssens W Jacquemin MG Saint-Remy JM. Increased synapse formation obtained by T cell epitopes containing a CxxC motif in flanking residues convert CD4+ T cells into cytolytic effectors. PLoS One (2012) 7:e45366. 10.1371/journal.pone.0045366 23056200 PMC3467281

[84] Malek Abrahimians E Carlier VA Vander Elst L Saint-Remy JM. MHC class II-restricted epitopes containing an oxidoreductase activity prompt CD4(+) T cells with apoptosis-inducing properties. Front Immunol (2015) 6:449. 10.3389/fimmu.2015.00449 26388872 PMC4556975

[85] Park Y Moon SJ Lee SW Lineage re-commitment of CD4CD8alphaalpha intraepithelial lymphocytes in the gut. BMB Rep (2016) 49:11–7. 10.5483/BMBRep.2016.49.1.242 26592937 PMC4914207

[86] Dutta A Huang CT Lin CY Chen TC Lin YC Chang CS Sterilizing immunity to influenza virus infection requires local antigen-specific T cell response in the lungs. Sci Rep (2016) 6:32973. 10.1038/srep32973 27596047 PMC5011745

[87] Law RH Zhang Q McGowan S Buckle AM Silverman GA Wong W An overview of the serpin superfamily. Genome Biol (2006) 7:216 10.1186/gb-2006-7-5-216 16737556 PMC1779521

[88] Rizzitelli A Meuter S Vega Ramos J Bird CH Mintern JD Mangan MS Serpinb9 (Spi6)-deficient mice are impaired in dendritic cell-mediated antigen cross-presentation. Immunol Cell Biol (2012) 90:841–51. 10.1038/icb.2012.29 22801574

[89] Münz C. Enhancing immunity through autophagy. Annu Rev Immunol (2009) 27:423–49. 10.1146/annurev.immunol.021908.132537 19105657

[90] Magnani CF Alberigo G Bacchetta R Serafini G Andreani M Roncarolo MG Killing of myeloid APCs via HLA class I, CD2 and CD226 defines a novel mechanism of suppression by human Tr1 cells. Eur J Immunol (2011) 41:1652–62. 10.1002/eji.201041120 21469116 PMC3116154

[91] Zeng H Zhang R Jin B Chen L. Type 1 regulatory T cells: a new mechanism of peripheral immune tolerance. Cell Mol Immunol (2015) 12:566–71. 10.1038/cmi.2015.44 26051475 PMC4579656

[92] Gregori S Goudy KS Roncarolo MG. The cellular and molecular mechanisms of immuno-suppression by human type 1 regulatory T cells. Front Immunol (2012) 3:30. 10.3389/fimmu.2012.00030 22566914 PMC3342353

[93] Gagliani N Magnani CF Huber S Gianolini ME Pala M Licona-Limon P Coexpression of CD49b and LAG-3 identifies human and mouse T regulatory type 1 cells. Nat Med (2013) 19:739–46. 10.1038/nm.3179 23624599

[94] Grossman W Verbsky J Tollefsen B Kemper C Atkinson J Ley T. Differential expression of granzymes A and B in human cytotoxic lymphocyte subsets and T regulatory cells. Blood (2004) 104:2840–8. 10.1182/blood-2004-03-0859 15238416

[95] Schmetterer KG Neunkirchner A Wojta-Stremayr D Leitner J Steinberger P Pickl WF. STAT3 governs hyporesponsiveness and granzyme B-dependent suppressive capacity in human CD4+ T cells. FASEB J (2015) 29:759–71. 10.1096/fj.14-257584 25398767 PMC4422363

[96] Debbabi H Ghosh S Kamath AB Alt J Demello DE Dunsmore S Primary type II alveolar epithelial cells present microbial antigens to antigen-specific CD4+ T cells. Am J Physiol Lung Cell Mol Physiol (2005) 289:L274–9. 10.1152/ajplung.00004.2005 15833765

[97] Gao J De BP Banerjee AK. Human parainfluenza virus type 3 up-regulates major histocompatibility complex class I and II expression on respiratory epithelial cells: involvement of a STAT1- and CIITA-independent pathway. J Virol (1999) 73:1411–8. 9882346 10.1128/jvi.73.2.1411-1418.1999 PMC103965

[98] Reith W Mach B. The bare lymphocyte syndrome and the regulation of MHC expression. Annu Rev Immunol (2001) 19:331–73. 10.1146/annurev.immunol.19.1.331 11244040

[99] Herkel J Jagemann B Wiegard C Lazaro JF Lueth S Kanzler S MHC class II-expressing hepatocytes function as antigen-presenting cells and activate specific CD4 T lymphocyutes. Hepatology (2003) 37:1079–85. 10.1053/jhep.2003.50191 12717388

[100] IARC Working Group on the Evaluation of Carcinogenic Risks to Humans. Biological agents. Volume 100 B. A review of human carcinogens. IARC Monogr Eval Carcinog Risks Hum (2012) 100:1–441. PMC4781184 23189750

[101] Nakagawa M Stites DP Patel S Farhat S Scott M Hills NK Persistence of human papillomavirus type 16 infection is associated with lack of cytotoxic T lymphocyte response to the E6 antigens. J Infect Dis (2000) 182:595–8. 10.1086/315706 10915094

[102] Garcia-Chagollan M Jave-Suarez LF Haramati J Bueno-Topete MR Aguilar-Lemarroy A Estrada-Chavez C An approach to the immunophenotypic features of circulating CD4(+)NKG2D(+) T cells in invasive cervical carcinoma. J Biomed Sci (2015) 22:91 10.1186/s12929-015-0190-7 26486970 PMC4615321

[103] Arce-Sillas A Alvarez-Luquin DD Tamaya-Dominguez B Gomez-Fuentes S Trejo-Garcia A Melo-Salas M Regulatory T cells: molecular actions on effector cells in immune regulation. J Immunol Res (2016) 2016:1720827. 10.1155/2016/1720827 27298831 PMC4889823

[104] Gondek D Lu L Quezada S Sakaguchi S Noelle R. Cutting edge: contact-mediated suppression by CD4+CD25+ regulatory cells involves a granzyme B-dependent, perforin-independent mechanism. J Immunol (2005) 174:1783–6. 10.4049/jimmunol.174.4.1783 15699103

[105] Loebbermann J Thornton H Durant L Sparwasser T Webster KE Sprent J Regulatory T cells expressing granzyme B play a critical role in controlling lung inflammation during acute viral infection. Mucosal Immunol (2012) 5:161–72. 10.1038/mi.2011.62 22236998 PMC3282434

[106] Efimova OV Kelley TW. Induction of granzyme B expression in T-cell receptor/CD28-stimulated human regulatory T cells is suppressed by inhibitors of the PI3K-mTOR pathway. BMC Immunol (2009) 10:59. 10.1186/1471-2172-10-59 19930596 PMC2784757

[107] Grossman WJ Verbsky JW Barchet W Colonna M Atkinson JP Ley TJ. Human T regulatory cells can use the perforin pathway to cause autologous target cell death. Immunity (2004) 21:589–601. 10.1016/j.immuni.2004.09.002 15485635

[108] Velaga S Ukena SN Dringenberg U Alter C Pardo J Kershaw O Granzyme A is required for regulatory T-cell mediated prevention of gastrointestinal graft-versus-host disease. PLoS One (2015) 10:e0124927. 10.1371/journal.pone.0124927 25928296 PMC4415808

[109] Cai SF Cao X Hassan A Fehniger TA Ley TJ. Granzyme B is not required for regulatory T cell-mediated suppression of graft-versus-host disease. Blood (2010) 115:1669–77. 10.1182/blood-2009-07-233676 19965675 PMC2832813

[110] Azzi J Skartsis N Mounayar M Magee CN Batal I Ting C Serine protease inhibitor 6 plays a critical role in protecting murine granzyme B-producing regulatory T cells. J Immunol (2013) 191:2319–27. 10.4049/jimmunol.1300851 23913965 PMC3750098

[111] Czystowska M Strauss L Bergmann C Szajnik M Rabinowich H Whiteside T. Reciprocal granzyme/perforin-mediated death of human regulatory and responder T cells is regulated by interleukin-2 (IL-2). J Mol Med (2010) 88:577–88. 10.1007/s00109-010-0602-9 20225066 PMC3777742

[112] Ashley C Baecher-Allan C. Cutting edge: responder T cells regulate human DR+ effector regulatory T cell activity via granzyme B. J Immunol (2009) 183:4843–7. 10.4049/jimmunol.0900845 19801510 PMC2918804

[113] von Gegerfelt A Valentin A Alicea C Van Rompay KK Marthas ML Montefiori DC Emergence of simian immunodeficiency virus-specific cytotoxic CD4+ T cells and increased humoral responses correlate with control of rebounding viremia in CD8-depleted macaques infected with Rev-independent live-attenuated simian immunodeficiency virus. J Immunol (2010) 185:3348–58. 10.4049/jimmunol.1000572 20702730 PMC7316374

[114] Sacha J Giraldo-Vela J Buechler M Martins M Maness N Chung C Gag- and Nef-specific CD4+ T cells recognize and inhibit SIV replication in infected macrophages early after infection. Proc Natl Acad Sci U S A (2009) 106:9791–6. 10.1073/pnas.0813106106 19478057 PMC2687996

[115] Couturier J Hutchison AT Medina MA Gingaras C Urvil P Yu X HIV replication in conjunction with granzyme B production by CCR5+ memory CD4 T cells: implications for bystander cell and tissue pathologies. Virology (2014) 46(2–463):175–88. 10.1016/j.virol.2014.06.008 24999042 PMC4158656

[116] Ayala VI Trivett MT Coren LV Jain S Bohn PS Wiseman RW A novel SIV gag-specific CD4(+)T-cell clone suppresses SIVmac239 replication in CD4(+)T cells revealing the interplay between antiviral effector cells and their infected targets. Virology (2016) 493:100–12. 10.1016/j.virol.2016.03.013 27017056 PMC4860118

[117] Zaunders JJ Munier ML Kaufmann DE Ip S Grey P Smith D Early proliferation of CCR5(+) CD38(+++) antigen-specific CD4(+) Th1 effector cells during primary HIV-1 infection. Blood (2005) 106:1660–7. 10.1182/blood-2005-01-0206 15905189

[118] Zheng N Fujiwara M Ueno T Oka S Takiguchi M. Strong ability of Nef-specific CD4+ cytotoxic T cells to suppress human immunodeficiency virus type 1 (HIV-1) replication in HIV-1-infected CD4+ T cells and macrophages. J Virol (2009) 83:7668–77. 10.1128/JVI.00513-09 19457989 PMC2708625

[119] Rosenberg ES Billingsley JM Caliendo AM Boswell SL Sax PE Kalams SA Vigorous HIV-1-specific CD4+ T cell responses associated with control of viremia. Science (1997) 278:1447–50. 10.1126/science.278.5342.1447 9367954

[120] Wang B Dyer WB Zaunders JJ Mikhail M Sullivan JS Williams L Comprehensive analyses of a unique HIV-1-infected nonprogressor reveal a complex association of immunobiological mechanisms in the context of replication-incompetent infection. Virology (2002) 304:246–64. 10.1006/viro.2002.1706 12504566

[121] Zaunders J van Bockel D. Innate and adaptive immunity in long-term non-progression in HIV disease. Front Immunol (2013) 4:95. 10.3389/fimmu.2013.00095 23630526 PMC3633949

[122] Harcourt GC Garrard S Davenport MP Edwards A Phillips RE HIV-1 variation diminishes CD4 T lymphocyte recognition. J Exp Med (1998) 188:1785–93. 10.1084/jem.188.10.1785 9815256 PMC2212407

[123] Burwitz BJ Giraldo-Vela JP Reed J Newman LP Bean AT Nimityongskul FA CD8+ and CD4+ cytotoxic T cell escape mutations precede breakthrough SIVmac239 viremia in an elite controller. Retrovirology (2012) 9:91. 10.1186/1742-4690-9-91 23131037 PMC3496649

[124] McKinstry KK Dutton RW Swain SL Strutt TM. Memory CD4 T cell-mediated immunity against influenza A virus: more than a little helpful. Arch Immunol Ther Exp (Warsz) (2013) 61:341–53. 10.1007/s00005-013-0236-z 23708562 PMC3874125

[125] Lukacher A Morrison L Braciale V Malissen B Braciale T. Expression of specific cytolytic activity by H-2I region-restricted, influenza virus-specific T lymphocyte clones. J Exp Med (1985) 162:171–87. 10.1084/jem.162.1.171 2409206 PMC2187708

[126] Zhou X McElhaney JE. Age-related changes in memory and effector T cells responding to influenza A/H3N2 and pandemic A/H1N1 strains in humans. Vaccine (2011) 29:2169–77. 10.1016/j.vaccine.2010.12.029 21353149 PMC3057414

[127] Zhang W Brahmakshatriya V Swain SL. CD4 T cell defects in the aged: causes, consequences and strategies to circumvent. Exp Gerontol (2014) 54:67–70. 10.1016/j.exger.2014.01.002 24440384 PMC3989398

[128] Gamadia LE Remmerswaal EB Weel JF Bemelman F van Lier RA ten Berge IJ. Primary immune responses to human CMV: a critical role for IFN-gamma-producing CD4+ T cells in protection against CMV disease. Blood (2003) 101:2686–92. 10.1182/blood-2002-08-2502 12411292

[129] van Leeuwen EM Remmerswaal EB Heemskerk MH Ten Berge IJ van Lier RA Strong selection of virus-specific cytotoxic CD4+ T cell clones during primary human cytomegalovirus infection. Blood (2006) 108(9):3121–7. 10.1182/blood-2006-03-006809 16840731

[130] Pachnio A Ciaurriz M Begum J Lal N Zuo J Beggs A Cytomegalovirus infection leads to development of high frequencies of cytotoxic virus-specific CD4+ T cells targeted to vascular endothelium. PLoS Pathog (2016) 12:e1005832. 10.1371/journal.ppat.1005832 27606804 PMC5015996

[131] Raeiszadeh M Pachnio A Begum J Craddock C Moss P Chen FE Characterization of CMV-specific CD4+ T-cell reconstitution following stem cell transplantation through the use of HLA class II-peptide tetramers identifies patients at high risk of recurrent CMV reactivation. Haematologica (2015) 100:e318–22. 10.3324/haematol.2015.123687 25975839 PMC5004434

[132] Shabir S Smith H Kaul B Pachnio A Jham S Kuravi S Cytomegalovirus-associated CD4(+) CD28(null) cells in NKG2D-dependent glomerular endothelial injury and kidney allograft dysfunction. Am J Transplant (2016) 16:1113–28. 10.1111/ajt.13614 26603521

[133] Jonjic S Mutter W Weiland F Reddehase MJ Koszinowski UH. Site-restricted persistent cytomegalovirus infection after selective long-term depletion of CD4+ T lymphocytes. J Exp Med (1989) 169:1199–212. 10.1084/jem.169.4.1199 2564415 PMC2189231

[134] Walton SM Mandaric S Torti N Zimmermann A Hengel H Oxenius A. Absence of cross-presenting cells in the salivary gland and viral immune evasion confine cytomegalovirus immune control to effector CD4 T cells. PLoS Pathog (2011) 7:e1002214. 10.1371/journal.ppat.1002214 21901102 PMC3161985

[135] Jeitziner SM Walton SM Torti N Oxenius A. Adoptive transfer of cytomegalovirus-specific effector CD4+ T cells provides antiviral protection from murine CMV infection. Eur J Immunol (2013) 43:2886–95. 10.1002/eji.201343690 23921569

[136] Verma S Weiskopf D Gupta A McDonald B Peters B Sette A Cytomegalovirus-specific CD4 T cells are cytolytic and mediate vaccine protection. J Virol (2016) 90:650–8. 10.1128/JVI.02123-15 PMC4702662 26491148

[137] Cohen JI Epstein-Barr virus infection. N Engl J Med (2000) 343:481–92. 10.1056/NEJM200008173430707 10944566

[138] Cohen JI Fauci AS Varmus H Nabel GJ. Epstein-Barr virus: an important vaccine target for cancer prevention. Sci Transl Med (2011) 3:107fs7. 10.1126/scitranslmed.3002878 22049067 PMC3501269

[139] Misko IS Pope JH Hutter R Soszynski TD Kane RG. HLA-DR-antigen-associated restriction of EBV-specific cytotoxic T-cell colonies. Int J Cancer (1984) 33:239–43. 10.1002/ijc.2910330212 6319303

[140] Stuller K Flano E. CD4 T cells mediate killing during persistent gammaherpesvirus 68 infection. J Virol (2009) 83:4700–3. 10.1128/JVI.02240-08 19244319 PMC2668489

[141] Hu Z Blackman MA Kaye KM Usherwood EJ Functional heterogeneity in the CD4+ T cell response to murine gamma-herpesvirus 68. J Immunol (2015) 194:2746–56. 10.4049/jimmunol.1401928 25662997 PMC4355211

[142] Long HM Haigh TA Gudgeon NH Leen AM Tsang CW Brooks J CD4+ T-cell responses to Epstein-Barr virus (EBV) latent-cycle antigens and the recognition of EBV-transformed lymphoblastoid cell lines. J Virol (2005) 79:4896–907. 10.1128/JVI.79.8.4896-4907.2005 15795275 PMC1069546

[143] Long HM Leese AM Chagoury OL Connerty SR Quarcoopome J Quinn LL Cytotoxic CD4+ T cell responses to EBV contrast with CD8 responses in breadth of lytic cycle antigen choice and in lytic cycle recognition. J Immunol (2011) 187:92–101. 10.4049/jimmunol.1100590 21622860 PMC3154640

[144] Nikiforow S Bottomly K Miller G Munz C. Cytolytic CD4(+)-T-cell clones reactive to EBNA1 inhibit Epstein-Barr virus-induced B-cell proliferation. J Virol (2003) 77:12088–104. 10.1128/JVI.77.22.12088-12104.2003 14581546 PMC254269

[145] Levitskaya J Coram M Levitsky V Imreh S Steigerwald-Mullen PM Klein G Inhibition of antigen processing by the internal repeat region of the Epstein-Barr virus nuclear antigen-1. Nature (1995) 375:685–8. 10.1038/375685a0 7540727

[146] Leung CS Haigh TA Mackay LK Rickinson AB Taylor GS. Nuclear location of an endogenously expressed antigen, EBNA1, restricts access to macroautophagy and the range of CD4 epitope display. Proc Natl Acad Sci U S A (2010) 107:2165–70. 10.1073/pnas.0909448107 20133861 PMC2836662

[147] Tagawa T Albanese M Bouvet M Moosmann A Mautner J Heissmeyer V Epstein-Barr viral miRNAs inhibit antiviral CD4+ T cell responses targeting IL-12 and peptide processing. J Exp Med (2016) 213:2065–80. 10.1084/jem.20160248 27621419 PMC5030804

[148] Adhikary D Behrends U Moosmann A Witter K Bornkamm GW Mautner J. Control of Epstein-Barr virus infection in vitro by T helper cells specific for virion glycoproteins. J Exp Med (2006) 203:995–1006. 10.1084/jem.20051287 16549597 PMC2118290

[149] Heller K Gurer C Munz C. Virus-specific CD4+ T cells: ready for direct attack. J Exp Med (2006) 203:805–8. 10.1084/jem.20060215 16549599 PMC2118265

[150] Akhmetzyanova I Zelinskyy G Schimmer S Brandau S Altenhoff P Sparwasser T Tumor-specific CD4+ T cells develop cytotoxic activity and eliminate virus-induced tumor cells in the absence of regulatory T cells. Cancer Immunol Immunother (2013) 62:257–71. 10.1007/s00262-012-1329-y 22890822 PMC3569596

[151] Akhmetzyanova I Zelinskyy G Littwitz-Salomon E Malyshkina A Dietze KK Streeck H CD137 agonist therapy can reprogram regulatory T cells into cytotoxic CD4+ T cells with antitumor activity. J Immunol (2016) 196:484–92. 10.4049/jimmunol.1403039 26608920

[152] Leskowitz R Fogg MH Zhou XY Kaur A Silveira EL Villinger F Adenovirus-based vaccines against rhesus lymphocryptovirus EBNA-1 induce expansion of specific CD8+ and CD4+ T cells in persistently infected rhesus macaques. J Virol (2014) 88:4721–35. 10.1128/JVI.03744-13 24522914 PMC3993789

[153] Taylor GS Jia H Harrington K Lee LW Turner J Ladell K A recombinant modified vaccinia ankara vaccine encoding Epstein-Barr virus (EBV) target antigens: a phase I trial in UK patients with EBV-positive cancer. Clin Cancer Res (2014) 20:5009–22. 10.1158/1078-0432.CCR-14-1122-T 25124688 PMC4340506

[154] Hui EP Taylor GS Jia H Ma BB Chan SL Ho R Phase I trial of recombinant modified vaccinia ankara encoding Epstein-Barr viral tumor antigens in nasopharyngeal carcinoma patients. Cancer Res (2013) 73:1676–88. 10.1158/0008-5472.CAN-12-2448 23348421 PMC6485495

[155] Weiskopf D Bangs DJ Sidney J Kolla RV De Silva AD de Silva AM Dengue virus infection elicits highly polarized CX3CR1+ cytotoxic CD4+ T cells associated with protective immunity. Proc Natl Acad Sci U S A (2015) 112:E4256–63. 10.1073/pnas.1505956112 26195744 PMC4534238

[156] Zhang Y Liu Y Moxley KM Golden-Mason L Hughes MG Liu T Transduction of human T cells with a novel T-cell receptor confers anti-HCV reactivity. PLoS Pathog (2010) 6:e1001018. 10.1371/journal.ppat.1001018 20686664 PMC2912399

[157] Fang M Siciliano NA Hersperger AR Roscoe F Hu A Ma X Perforin-dependent CD4+ T-cell cytotoxicity contributes to control a murine poxvirus infection. Proc Natl Acad Sci U S A (2012) 109:9983–8. 10.1073/pnas.1202143109 22665800 PMC3382508

[158] Siciliano NA Hersperger AR Lacuanan AM Xu RH Sidney J Sette A Impact of distinct poxvirus infections on the specificities and functionalities of CD4+ T cell responses. J Virol (2014) 88:10078–91. 10.1128/JVI.01150-14 24965457 PMC4136331

[159] Littaua RA Takeda A Cruz J Ennis FA Vaccinia virus-specific human CD4+ cytotoxic T-lymphocyte clones. J Virol (1992) 66:2274–80. 1548761 10.1128/jvi.66.4.2274-2280.1992 PMC289021

[160] Demkowicz WE Jr Littaua RA Wang J Ennis FA. Human cytotoxic T-cell memory: long-lived responses to vaccinia virus. J Virol (1996) 70:2627–31. 8642697 10.1128/jvi.70.4.2627-2631.1996 PMC190113

[161] Mitra-Kaushik S Cruz J Stern LJ Ennis FA Terajima M. Human cytotoxic CD4+ T cells recognize HLA-DR1-restricted epitopes on vaccinia virus proteins A24R and D1R conserved among poxviruses. J Immunol (2007) 179:1303–12. 10.4049/jimmunol.179.2.1303 17617623

[162] Heegaard ED Brown KE. Human parvovirus B19. Clin Microbiol Rev (2002) 15:485–505. 10.1128/CMR.15.3.485-505.2002 12097253 PMC118081

[163] Watson AM Lam LK Klimstra WB Ryman KD. The 17D-204 vaccine strain-induced protection against virulent yellow fever virus is mediated by humoral immunity and CD4+ but not CD8+ T cells. PLoS Pathog (2016) 12:e1005786. 10.1371/journal.ppat.1005786 27463517 PMC4962991

[164] Vogel AJ Brown DM. Single-dose CpG immunization protects against a heterosubtypic challenge and generates antigen-specific memory T cells. Front Immunol (2015) 6:327. 10.3389/fimmu.2015.00327 26161083 PMC4479795

[165] Babon JA Cruz J Ennis FA Yin L Terajima M. A human CD4+ T cell epitope in the influenza hemagglutinin is cross-reactive to influenza A virus subtypes and to influenza B virus. J Virol (2012) 86:9233–43. 10.1128/JVI.06325-11 22718815 PMC3416118

[166] McElhaney JE Coler RN Baldwin SL. Immunologic correlates of protection and potential role for adjuvants to improve influenza vaccines in older adults. Expert Rev Vaccines (2013) 12:759–66. 10.1586/14760584.2013.811193 23885821

[167] Terahara K Ishii H Nomura T Takahashi N Takeda A Shiino T Vaccine-induced CD107a+ CD4+ T cells are resistant to depletion following AIDS virus infection. J Virol (2014) 88:14232–40. 10.1128/JVI.02032-14 25275131 PMC4249136

[168] Haynes BF Gilbert PB McElrath MJ Zolla-Pazner S Tomaras GD Alam SM Immune-correlates analysis of an HIV-1 vaccine efficacy trial. N Engl J Med (2012) 366:1275–86. 10.1056/NEJMoa1113425 22475592 PMC3371689

[169] Lin L Finak G Ushey K Seshadri C Hawn TR Frahm N COMPASS identifies T-cell subsets correlated with clinical outcomes. Nat Biotechnol (2015) 33:610–6. 10.1038/nbt.3187 26006008 PMC4569006

